# Exploring the Role of Anionic Lipid Nanodomains in the Membrane Disruption and Protein Folding of Human Islet Amyloid Polypeptide Oligomers on Lipid Membrane Surfaces Using Multiscale Molecular Dynamics Simulations

**DOI:** 10.3390/molecules28104191

**Published:** 2023-05-19

**Authors:** Ngoc Nguyen, Amber Lewis, Thuong Pham, Donald Sikazwe, Kwan H. Cheng

**Affiliations:** 1 Physics Department, Trinity University, San Antonio, TX 78212, USA; nnguyen5@trinity.edu (N.N.); tpham3@trinity.edu (T.P.); 2 Neuroscience Department, Trinity University, San Antonio, TX 78212, USA; alewis2@trinity.edu; 3 Pharmaceutical Sciences Department, Feik School of Pharmacy, University of the Incarnate Word, San Antonio, TX 78209, USA; sikazwe@uiwtx.edu

**Keywords:** intrinsically disordered protein, lipid nanodomains, surface nanostructures, protein–lipid interaction, protein folding, multiscale molecular modeling, computational physical chemistry

## Abstract

The aggregation of human Islet Amyloid Polypeptide (hIAPP) on cell membranes is linked to amyloid diseases. However, the physio-chemical mechanisms of how these hIAPP aggregates trigger membrane damage are unclear. Using coarse-grained and all-atom molecular dynamics simulations, we investigated the role of lipid nanodomains in the presence or absence of anionic lipids, phosphatidylserine (PS), and a ganglioside (GM1), in the membrane disruption and protein folding behaviors of hIAPP aggregates on phase-separated raft membranes. Our raft membranes contain liquid-ordered (Lo), liquid-disordered (Ld), mixed Lo/Ld (Lod), PS-cluster, and GM1-cluster nanosized domains. We observed that hIAPP aggregates bound to the Lod domain in the absence of anionic lipids, but also to the GM1-cluster- and PS-cluster-containing domains, with stronger affinity in the presence of anionic lipids. We discovered that L16 and I26 are the lipid anchoring residues of hIAPP binding to the Lod and PS-cluster domains. Finally, significant lipid acyl chain order disruption in the annular lipid shells surrounding the membrane-bound hIAPP aggregates and protein folding, particularly beta-sheet formation, in larger protein aggregates were evident. We propose that the interactions of hIAPP and both non-anionic and anionic lipid nanodomains represent key molecular events of membrane damage associated with the pathogenesis of amyloid diseases.

## 1. Introduction

The misfolding and subsequent self-aggregation of intrinsically disordered proteins, such as human Islet Amyloid Polypeptide (hIAPP), on the surfaces of lipid membranes are the major molecular mechanisms leading to the progression of various amyloid diseases [1]. However, how the physio-chemical properties of the phase-separated lipid nanodomains, or raft membrane domains, of different nanostructures modulate and trigger protein-induced membrane damage upon protein binding to the cell membrane are still unclear. Experimentally, two-step membrane damage mechanisms involving the formation of ion pores and membrane fragmentation due to surface-induced fibril growth [2,3,4,5,6] have been proposed. Yet, the early and detailed molecular events associated with the binding of disordered hIAPP aggregates from the solution state to the membrane-bound state that trigger membrane damage are not fully understood. In addition, how these disordered aggregates interact with a realistic lipid membrane model involving the presence of the highly dynamic and heterogeneous lipid domains of different nanostructures, or lipid raft domains, has not been fully explored. Note that the dynamic, phase-separated lipid nanodomains have been experimentally confirmed on both model and biological membranes [7,8,9,10,11,12,13,14]. Furthermore, the physio-chemical properties and functional roles of lipid nanodomains have been linked to the progression of amyloid diseases, such as type 2 diabetes and various neurodegenerative diseases [13,14]. 

To explore the molecular mechanisms of hIAPP-induced membrane damage, we have designed a 37-residue-long disordered hIAPP monomer [15,16]. Disordered monomers, and self-aggregated dimers and tetramers, or hIAPP oligomers, in solution were subsequently constructed using a coarse-grained (CG) model [16]. For the lipid membrane, we have designed several multi-component phase-separated lipid bilayers, or raft membranes, containing dynamic lipid nanodomains as our lipid membrane model to investigate protein-induced membrane damage by disordered hIAPP oligomers on lipid nanodomains of different nanostructures [17,18]. 

Most previous computational and experimental studies [1,19,20,21,22,23] on hIAPP–lipid interactions have been focused on homogeneous, multi-component lipid bilayer systems containing zwitterionic phosphatidylcholines (PCs); anionic PCs, such as phosphatidylserine (PS) and a ganglioside (GM1); and cholesterol (CHOL). The significant roles of lipid acyl chain unsaturation, headgroup charge, and CHOL in partially disordered and fibrillar hIAPP–lipid interactions and membrane permeabilization have been investigated in various lipid mixtures. Overall, hIAPP monomers or oligomers strongly bind to anionic lipids, partially because of the net +2e charge of hIAPP, and prefer unsaturated lipids over saturated lipids [6,24,25,26]. Experimentally, using a raft-like model membrane containing a zwitterionic PC similar to the raft membrane in this study, the presence of CHOL enhances membrane leakage with pore formation but suppresses fiber growth on membrane surfaces. However, in PS-containing membranes, CHOL inhibits pore formation but enhances fiber growth on membrane surfaces [27]. The presence of gangliosides has also been known to promote hIAPP binding and folding on membrane surfaces [28,29]. At present, the detailed molecular mechanisms of hIAPP–lipid nanodomain interactions at the atomistic level are still unclear. This study focuses on raft membranes containing both neutral lipid nanodomains and anionic lipid nanodomains involving PS and GM1. 

Using a CG membrane model, our model raft membrane consists of a three-component lipid bilayer (CO-raft) containing CHOL, a saturated PC, and an unsaturated PC, as well as two four-component lipid bilayers containing CHOL, saturated dipalmitoyl-PC (DPPC), unsaturated dilinoleoyl-PC (DLPC), and an anionic lipid. The raft membrane containing PS or GM1 is called PS-raft or GM-raft, respectively [17]. Under physiological conditions, highly dynamic and phase-separated liquid-ordered (Lo) domains, liquid-disordered (Ld) domains, and mixed Lo/Ld (Lod) domains are spontaneously formed on the microsecond time scale. These phase-separated Lo, Ld, and Lod domains mimic the ordered, cholesterol- and saturated PC-enriched raft region; the disordered, cholesterol-depleted, and unsaturated PC-enriched disordered non-raft region; and the boundary region containing both saturated and unsaturated PCs, and some cholesterol, respectively [30]. These Lo, Ld, and Lod lipid nanodomains are found on both the inner and outer leaflets of the model membranes and in biological cell membranes [17]. On the other hand, our PS-raft and GM-raft present PS-clusters or GM1-clusters on only one leaflet of the membranes [17]. Interestingly, PS and GM1 lipids are exclusively found on the inner leaflet and outer leaflets of the plasma membrane of all mammalian cells [31]. Therefore, our CO-raft, PS-raft, and GM-raft are physio-chemically and biologically relevant lipid nanodomains found on both lipid leaflets of the plasma membrane. The molecular interactions between hIAPP oligomers and these lipid nanodomains in the raft membrane were systematically examined using CG molecular dynamics (MD) simulations of up to 15 microseconds.

Although CG simulations of our hIAPP-raft systems allow us to examine the protein binding events and mechanisms on the microsecond scale, membrane-induced protein folding, or protein secondary structure transition, and detailed protein-induced membrane damage cannot be obtained with CG simulation alone. Here, we used CG-to-atomistic (AA) resolution transformation [32] followed by 300-nanosecond-long AA simulations to observe the membrane structural disruption and protein folding events of membrane-bound hIAPP oligomers in atomistic detail. 

Our combined CG and AA, or multiscale, approach allowed us to sample sufficient translational and rotational conformational space of protein–membrane binding events, establish the lipid binding kinetics and protein residue-resolved lipid binding patterns, and analyze detailed membrane structural disruption and protein folding events on the raft membranes of both neutral and anionic lipid domains. These results will be useful to guide new experiments on examining disordered hIAPP oligomer interactions with the phase-separated model and cell membranes. In addition, the secondary structure of the membrane-bound hIAPP oligomers will provide insights for the future development of protein-based drug design targeting the early membrane-bound structures of hIAPP oligomers on either the inner or outer leaflet of cell membranes. 

## 2. Results

Using multiscale, CG and AA, MD simulations, this study focused on investigating the *lipid binding behaviors*, *protein-induced membrane disruption*, and *protein folding* of human islet polypeptide oligomers [hIAPP]_n_ of various aggregation sizes, i.e., *n* = 1 (monomers), *n* = 2 (dimers), and *n* = 4 (tetramers), on three different raft membranes, i.e., CO-raft, PS-raft, and GM-raft. Our raft membranes contain phase-separated lipid nanodomains with and without asymmetrically distributed PS- and GM1-clusters. 

### 2.1. Initial Structures of [hIAPP]_n_ and Raft Membranes in Solution

The initial hIAPP monomer structure carries a net charge of +2e and contains a flexible *N* terminus and a collapsed, non-structured *C* terminus derived from a pentamer Cryo-EM structure (see Section 4). The initial hIAPP dimer and tetramer structures were created using a self-aggregation process of multiple hIAPP monomers in solution. Before binding to lipid membranes, all hIAPP oligomers were highly flexible and disordered. 

**Figure 1:**
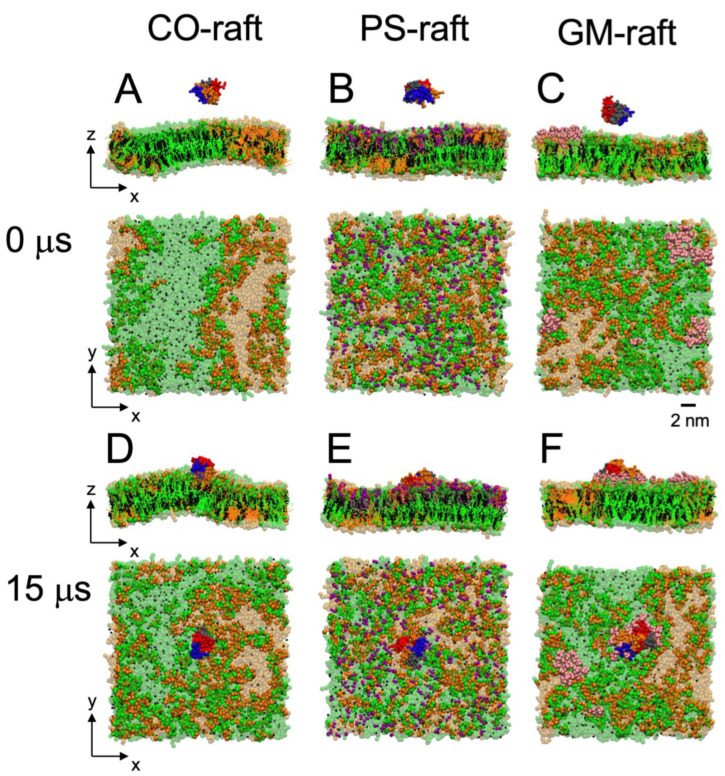
hIAPP tetramers binding to raft membranes. The beginning structures (0 μs) consist of the hIAPP tetramer placed ~ 5 nm above the center of the CO-raft (**A**), PS-raft (**B**), or GM-raft (**C**). The final structures (15 μs) consist of the membrane-bound hIAPP tetramer on the surfaces of the CO-raft (**D**), PS-raft (**E**), or GM-raft (**F**). DPPC lipids are in green, with DPPC-enriched domains (Lo) in a lighter color. DLPC lipids are in orange, with DLPC-enriched domains (Ld) in a lighter color. POPS lipids are in red, and GM1 lipids, in pink. The chains of tetramers are shown in color beads, with chain A in blue, chain B in red, chain C in gray, and chain D in orange. All simulations were performed in 0.1 M NaCl at 310 K under 1-atmosphere pressure in explicit solvent at coarse-grained resolution (See Section 4). Both the transverse (x-z) and lateral (x-y) views are shown. A scale bar of 2 nm is also given.

This study involved three raft membranes: CO-raft, PS-raft, and GM-raft. The lateral organization of lipid molecules on the top leaflet of each raft membrane before protein binding is demonstrated in the lateral views (*x*, *y*) in Figure 1A–C. Here, the top leaflet refers to the lipid leaflet facing the externally added protein before protein binding, and the bottom leaflet is the other lipid leaflet not facing the protein.

Both top and bottom leaflets exhibited highly dynamic and phase-separated lipid nanodomains or nanostructures. On the CO-raft, DPPC-rich Lo, DLPC-rich Ld, and mixed DPPC-DLPC (or Lod) nanodomains were observed on both the top and bottom leaflets of the lipid bilayer (Figure 1A). On the PS-raft, anionic and partially saturated 1-palmitoyl-2-oleoyl-PS (POPS) lipids partitioned to the Lod domain on the top leaflet of the bilayer (Figure 1B). On the GM-raft, two-to-three GM1-clusters partitioned to the Lo domain on the top leaflet of the bilayer (Figure 1C). The asymmetric, or leaflet-specific, distribution of PS-clusters or GM-clusters is further demonstrated in the transverse view of the PS-raft (Figure 1B) or GM-raft (Figure 1C). 

The three raft membranes have different charge properties. Since DPPC, DLPC, and CHOL are neutral lipids, both the top- and bottom-leaflet surfaces of the CO-raft contain no net charge. On the other hand, POPS and GM1 are anionic, or negatively charged, lipids; thus, the top-leaflet surface of both the PS-raft and the GM-raft is negatively charged. Therefore, our initial raft membranes consisted of a symmetric CO-raft, an asymmetric PS-raft, and an asymmetric GM-raft based on the differences in the *lateral organization* of lipids and *surface negative charge* distribution on the top and bottom leaflets of the raft membranes. 

Each initial simulation structure in this study consisted of a physically separated hIAPP oligomer of a given aggregation size and one type of raft membrane in solution (Figure 1A–C). Three independent simulation replicates, representing three different positions of the protein above the membrane surface, were generated for each simulation system (see Section 4). The transverse view (*x*-*z*) in Figure 1A–C demonstrates the vertical arrangement of an hIAPP tetramer above each raft membrane. Here, the *z*-direction is along the normal of the lipid bilayer plane (*x*-*y*). The other two replicates (not shown) were subsequently generated with the protein position shifted by ±2 nm along the *x*-direction relative to the position of the first replicate, as shown in Figure 1A–C. 

Overall, a total of 27 independent initial structures involving oligomers of three aggregation sizes, three raft membrane types, and three simulation replicates were created in this multiscale simulation study.

### 2.2. Kinetics and Lipid Domain Preference in [hIAPP]_n_ Binding to Raft Membranes

All hIAPP oligomers bound to the surfaces of the raft membranes from the solution state to a membrane-bound state during our 0-to-15 μs or longer CG simulations. Figure 1 demonstrates the transverse and lateral views of the attachment of an hIAPP tetramer to the CO-raft (Figure 1D), PS-raft (Figure 1E), and GM-raft (Figure 1F) from representative simulation replicates. 

In this study, the majority of the simulation replicates successfully bound to the top leaflet of the raft membranes. Here, among nine replicates, six replicates for the CO-raft, eight replicates for the PS-raft, and all nine replicates for the GM-raft bound to the upper leaflet. The replicate that bound to the bottom leaflet of the PS-raft was from an hIAPP monomer. A new simulation based on the same initial position but different initial velocity distribution of the hIAPP monomer above the surface of the PS-raft was performed, and a successful binding to the top leaflet was achieved. Since the CO-raft contains Lo, Ld, and Lod on both leaflets, all nine replicates bound to different leaflets were used. 

**Figure 2:**
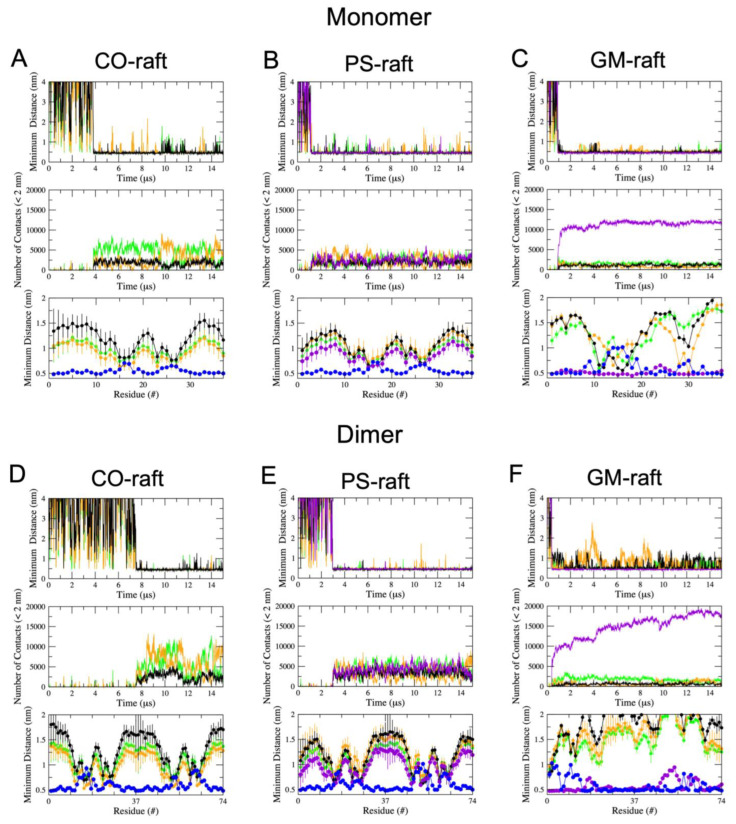
Protein–lipid and protein–water minimum distance analysis of hIAPP monomers and dimers binding to raft membranes. Three-panel plots of protein–lipid and protein–water minimum distance (*mindist*) of monomeric (**A**–**C**) and dimeric (**D**–**F**) hIAPP aggregates on the CO-raft (**A**,**D**), PS-raft (**B**,**E**), and GM-raft (**C**,**F**) are given. For each system, the upper panel shows *mindist* vs. time; the middle panel shows the number of contacts between protein and lipid atoms within 2 nm vs. time; and the lower panel shows the time-averaged mindist vs. protein residue number over the last 5 μs. All *mindist* data points are color-coded, with DPPC in green, DLPC in orange, CHOL in black, POPS or GM1 in purple, and water in blue.

**Figure 3:**
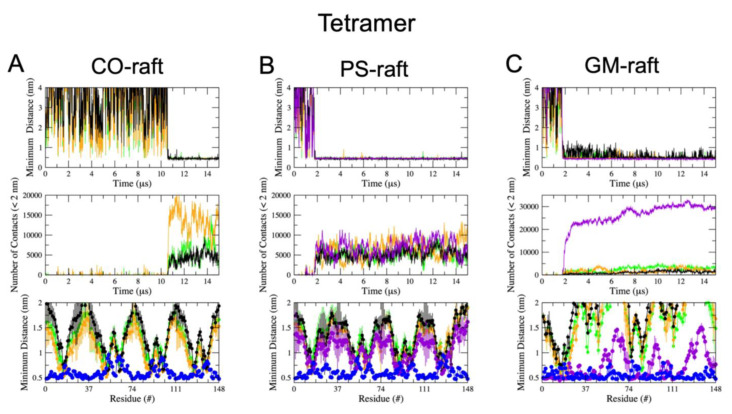
Protein–lipid and protein–water minimum distance analysis of hIAPP tetramers binding to raft membranes. Three-panel plots of protein–lipid and protein–water minimum distance (*mindist*) of tetrameric hIAPP aggregates on the CO-raft (**A**), PS-raft (**B**), and GM-raft (**C**) are given. All *mindist* data points are color-coded, with DPPC in green, DLPC in orange, CHOL in black, POPS or GM1 in purple, and water in blue. See the legend of Figure 2 for details.

We observed an interesting dependence of the binding kinetics of hIAPP oligomers on the nanostructure of the raft membranes. Table 1 summarizes the time of the lipid binding of the hIAPP oligomer to the raft surface based on the minimum distance (*mindist*) between the atoms of the protein and lipids vs. time (upper panel) of each replicate as demonstrated in Figure 2 and Figure 3. Here, the lipid binding time is defined as the time at which the protein undergoes an abrupt transition from the solution state with a large and fluctuating *mindist* value to a membrane-bound state with a small and stable *mindist* value. For example, abrupt declines in *mindist* were detected at 3.84, 1.19, and 0.94 μs for the hIAPP monomer on the CO-raft (Figure 2A), PS-raft (Figure 2B), and GM-raft (Figure 2C), respectively. From Table 1, it is evident that the lipid binding time was the longest with the CO-raft, with an average of ~5 μs, compared with ~2 μs relative to the PS-raft and ~0.7 μs relative to the GM-raft, across all simulation replicates and all aggregation sizes. From the lipid binding kinetic point of view, the hIAPP oligomer prefers to bind to the GM-raft followed by the PS-raft.

A major goal of this study was to identify the lipid domain preference of each hIAPP oligomer for the surface nanostructure of each raft membrane. Here, the *direct visualization* using VMD (see Section 4) of the protein/raft complex during CG simulations, *minimum distance analysis*, and *annular lipid analysis* were used to explore the lipid domain preference of hIAPP oligomers. 

For the direct visualization of lipid domain preference in protein binding, Figure 1D–F demonstrate the transverse and lateral structures of the membrane-bound tetramer bound to each raft membrane from a representative replicate at the conclusion of the 0–15 μs CG simulation. We observed the preferential binding of the protein to the Lod domain on the CO-raft (Figure 1D), the PS-cluster of the Lod domain on the PS-raft (Figure 1E), and the GM-cluster-containing Lo domain on the GM-raft. Similar observations among all replicates were found for oligomers of all sizes. 

The *mindist* analysis represents our second approach to exploring lipid domain preference in protein binding, as shown in Figure 2 and Figure 3. Here, the *mindist* between protein and different lipid types vs. time (upper panel) reveal that oligomers of all sizes, i.e., monomers, dimers, and tetramers, bound to all lipid components of each raft membrane, i.e., DPPC, DLPC, and CHOL, on the CO-raft; DPPC, DLPC, CHOL, and POPS on the PS-raft; and DPPC, DLPC, CHOL, and GM1, on the GM-raft. The fluctuation in the *mindist* values was evident in the mindist vs. time plots, reflecting the highly dynamic nature of protein–lipid interactions. The plots of the number of contacts between protein and lipid atoms within 2 nm vs. time (mid panel) further revealed the involvement of all lipid types in protein binding to the raft membranes.

Lastly, the annular lipid analysis provided the most quantitative approach to investigating lipid domain preference in protein binding. The results of the time- and replicate-averaged composition of the 0.5 nm annular lipid shells surrounding the membrane-bound oligomers in the CG simulations are given in Table 1. Regarding the CO-raft, the composition was ~20% CHOL, 30% DPPC, and 50% DLPC. Regarding the PS-raft, the composition was ~20% CHOL, 25% DPPC, 25% DLPC, and 30% POPS. Finally, regarding the GM-raft, the composition was ~5% CHOL, 10% DPPC, 5% DLPC, and 80% GM1. These composition results were independent of the size of the membrane-bound oligomers. 

The above direct visualization, *mindist* analysis, and annular lipid analysis, therefore, revealed that the hIAPP oligomers prefer the Lod domain on the CO-raft, the PS-clusters within the Lod domain, and the GM1-cluster within the Lo domain. 

Note that protein binding to raft membranes resulted in subtle changes in the nanodomain organization of the three raft membranes. Figure SA1 shows the lipid composition of Lo, Ld, and Lod on the raft membranes in the absence and presence of hIAPP oligomers. We found a small and progressive increase in the percentage of Lod-CHOL (Figure SA1A) or Lod-PC (Figure SA1B) with the increase in the size of the hIAPP oligomer on the CO-raft. In contrast, a small and progressive decrease in the percentage of Lod-CHOL (Figure SA1C) or Lod-PC (Figure SA1D) with the increase in the size of the hIAPP oligomer on the PS-raft was evident. Yet, no significant change in the percentage of Lo-DPPC (Figure SA1E) nor Lo-CHOL (Figure SA1F) was detected. 

In addition, the composition of larger annular lipid shells, i.e., 0.5–1, 1–2, and 2–3 nm away from the membrane-bound oligomer, was compared with the reported composition of the nearest (0.5 nm away) annular lipid shell, as shown in Table 1. Figure SA2 shows the number of lipids in all annular lipid shells of all lipid types in all three raft membranes. As expected, a progressive increase in the number of DPPC, DLPC, and CHOL lipids with the size of the annular lipid shell was found in all three raft membranes. However, the number of GM1 lipids was the highest in the 0.5 nm annular lipid shell compared with the other annular lipid shells (Figure SA2K), which is indicative of the preferential binding of the oligomers to the GM1-clusters on the GM-raft. 

From the numbers of lipids of different types, the percentages of different lipid types in each annular lipid shell were also evaluated (Figure SA3). In all three raft membrane types, CO-raft, PS-raft, and GM-raft, the percentage of CHOL or DPPC progressively increased with the size of the annular lipid shell. A very different trend was found for the percentage of DLPC. The DLPC percentage decreased, remained relatively the same, and increased in the CO-raft (Figure SA3C), PS-raft (Figure SA3G), and GM-raft (Figure SA3J), respectively. Interestingly, the percentage of POPS in the PS-raft or GM1 in the GM1-raft decreased with the size of the annular lipid shell, which is indicative of the enrichment of POPS or GM1 in the nearest annular lipid shell surrounding the membrane-bound protein. Due to the highly dynamic nature of raft membranes, large fluctuations in the numbers of lipids within the smallest 0.5 nm annular lipid shell were evident in both CG and AA simulations, as demonstrated in Figure SA4.

### 2.3. Residue-Specific Protein–Lipid Binding Pattern of [hIAPP]_n_ on Raft Membrane Surfaces

**Figure 4:**
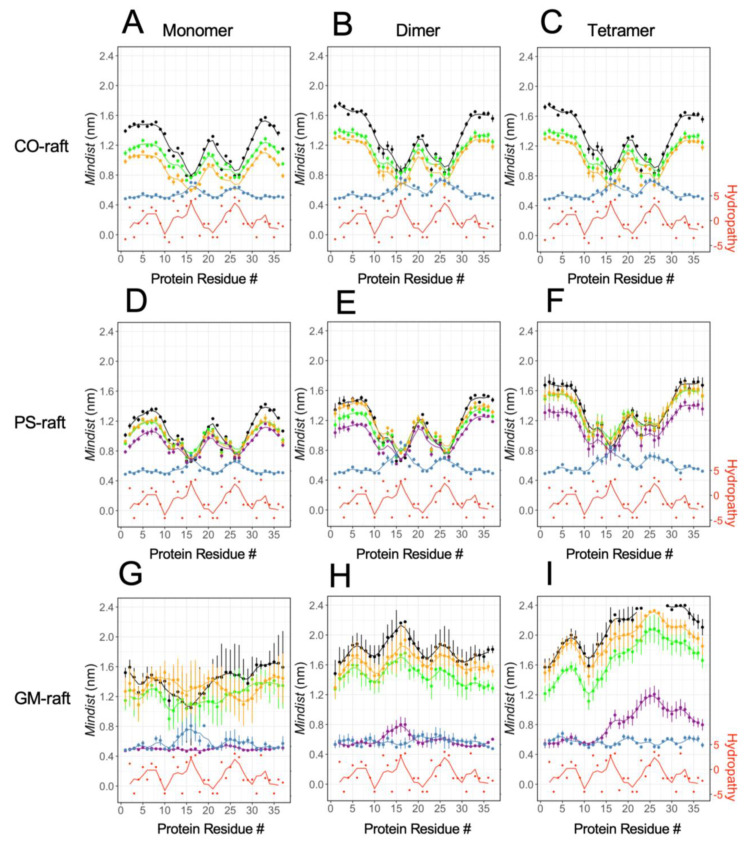
Protein–lipid and protein–water minimum distance spectra of hIAPP aggregates on raft membranes. The minimum distance (*mindist*) spectrum is defined as the time-, replicate- and chain-averaged minimum distance between protein and lipid (or water) atoms vs. protein residue of membrane-bound hIAPP monomer (**A**,**D**,**G**), dimer (**B**,**E**,**H**), and tetramer (**C**,**F**,**I**) on the CO-raft (**A**–**C**), PS-raft (**D**–**F**), and GM-raft (**G**–**I**) in CG simulations. Lipid molecules, DPPC, DLPC, CHOL, and POPS (or GM1), are labeled in green, orange, black, and purple, respectively. Water is labeled in blue. In addition, the hydropathy index vs. protein residue plot labeled in red on the secondary (right-side) y-axis was added to facilitate the interpretation of the residue specificity of protein binding to lipid or water. A 3-point moving average fit was applied to the mindist spectra and hydropathy plots to quantify the peaks or dips of the plots. The error bar represents the standard error of the means for each *mindist* value.

Upon establishing the kinetics of and nanodomain preference in protein binding, we proceeded to explore the pattern of residue-specific binding of hIAPP oligomers to the different nanostructures of each raft membrane. The lower panel of each three-panel plot in Figure 2 and Figure 3 shows the time-averaged *mindist* vs. protein residue plot of the hIAPP oligomer binding to the raft membrane. Significant dips, which are indicative of the involvement of specific protein residues interacting with different lipid types, in each raft membrane were evident. To further explore the binding patterns, the time-, replicate-, and chain-averaged mindist vs. residue plot of hIAPP oligomers of a given size for each raft membrane was calculated, and the results are summarized in Figure 4.

A consistent residue-specific protein–lipid binding pattern emerged in the triple-averaged *mindist* vs. protein residue number plots for the CO-raft (Figure 4A–C) and PS-raft (Figure 4D–F). Here, major dips were discovered at residues #16 and #26 of hIAPP oligomers of all sizes. In addition, the *mindist* values followed the ranking orders of DLPC < DPPC < CHOL for the CO-raft and POPS < DPPC ~ DLPC < CHOL for the PS-raft. Note that the *mindist* value of DPPC was slightly lower than that of DLPC in the lower residue region, or *N* terminus, of the dimer (Figure 4E), but no differences among the DPPC and DLPC *mindist* values were detected in none of the residues of the monomer (Figure 4D) or the tetramer (Figure 4F). 

Regarding the GM-raft, the residue-specific binding pattern exhibited interesting oligomer size-dependent behavior, as shown in Figure 4G–I. Here, the dips of the GM1 *mindist* values were constant at around 0.5 nm, with no dips for the monomer (Figure 4G). As the size of the oligomer increased, a major peak, instead of a dip, at residue #16 was evident for the dimer (Figure 4H), and the *mindist* peak shifted to residue #26 of the tetramer (Figure 4I). Regarding the other lipid types, i.e., DLPC, DPPC, and CHOL, the overall *mindist* values were significantly higher than those of lipids in the CO-raft or PS-raft, which is indicative of a farther distance between non-GM1 lipids and the bound protein in the GM-raft. 

In addition to the protein–lipid *mindist* values, the protein–water *mindist* value and the hydropathy values (see Section 4) are also presented. As described in Methods, the peaks of the protein–water *mindist* vs. protein residue corresponded to the regions of the protein that were void of water contact, while the peaks of the hydropathy plots indicated the hydrophobic regions of the protein. Here, the above residues, #16 and #26, matched the two hydrophobic peak regions of hIAPP and the protein–water *mindist* peak regions for the CO-raft and PS-raft, as shown in Figure 4, which is indicative of the hydrophobic interactions between residues #16 and #26 of the protein, and the lipids. Regarding the GM-raft, the observed major *mindist* peak at residue #16 of the dimer and the broad *mindist* peak at residue #26 of the tetramer indicated that the protein avoided contacts with GM1 lipids at those hydrophobic residues. In other words, the protein–lipid interactions on the GM-raft were mainly hydrophilic with hIAPP oligomers, particularly with the tetramer. 

Similar protein–lipid and protein–water binding patterns obtained from the *mindist* vs. protein residue number plots were observed at AA resolution, and the results are given in Figure SB1. Here, dips at residues #16 and #26 of all oligomers on the CO-raft and PS-raft, and distinctive peaks at residues #16 and #26 of the dimers and tetramers on the GM-raft were also detected. 

**Figure 5:**
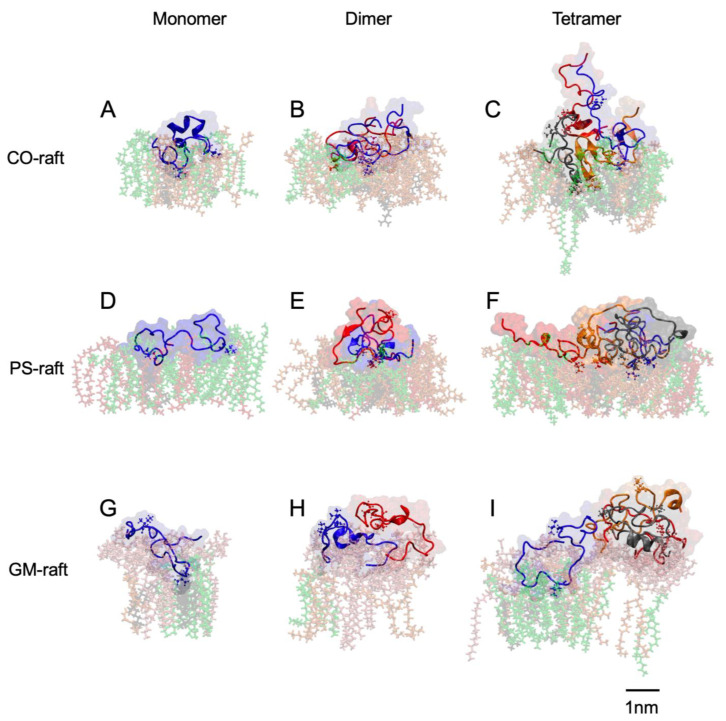
Structures of membrane-bound hIAPP aggregates bound to raft membranes. The transverse views of hIAPP monomer (**A**,**D**,**G**), dimer (**B**,**E**,**H**), and tetramer (**C**,**F**,**I**), and the surrounding lipids in 0.5 nm annular lipid shells. The protein structure is shown in both colored all-atom surface and peptide backbone ribbon forms, with chain A in blue, chain B in red, chain C in gray, and chain D in orange. In addition, the two hydrophobic residues at L16 and I 26 of each protein chain are shown in licorice. Lipid molecules, DPPC, DLPC, CHOL, POPS, and GM1, in the annular lipid shell are labeled in light green, orange, black, red, and pink, respectively. A scale bar of 1 nm is also shown.

To visualize the residue-resolved patterns of protein binding to the raft membranes, Figure 5 demonstrates the AA structures of the membrane-bound oligomers of different sizes on the CO-raft (Figure 5A–C), PS-raft (Figure 5D–F), and GM-raft (Figure 5G–I) from representative replicates. The AA structures of all replicates for the CO-raft, PS-raft, and GM-raft are given in Figures SC1–SC3, respectively. Here, the 0.5 nm annular lipid shells surrounding the bound protein are shown to illustrate the coupling between hIAPP and the lipid molecules of different types upon protein binding. Residues #16 and #26 were identified in all the AA structures. From these membrane-bound oligomers and annular shell structures, it was evident that residues #16 and #26 preferentially deeply bound to the lipid membranes of the CO-raft and PS-raft. Except for the monomer, most residues #16 and #26 were exposed outside the membrane surface of the GM-raft in agreement with the *mindist* vs. protein residue analysis.

### 2.4. Binding Energies of Membrane-Bound [hIAPP]_n_

The protein–lipid and protein–protein binding energies associated with the hIAPP oligomers bound to different raft membrane surfaces were investigated at both CG and AA resolution. 

**Figure 6:**
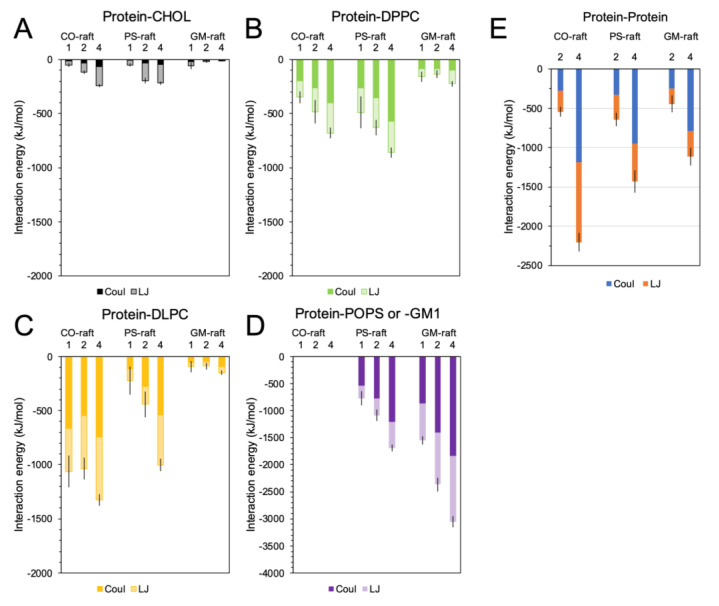
Protein–lipid and protein–protein interaction energies of hIAPP aggregates on raft membranes. Protein–lipid and protein–protein interaction energies of membrane-bound hIAPP aggregates of sizes *n* = 1 (monomer), 2 (dimer), and 4 (tetramer), on CO-, PS-, and GM-rafts for different lipid types, i.e., CHOL (**A**), DPPC (**B**), DLPC (**C**), and GM1 or POPS (**D**), and among adjacent protein chains (**E**) are shown. Both Coulomb (darker) and Lennard-Jones (light) interaction energies for each lipid type and among adjacent protein chains are highlighted. Each data point represents the time and replicate average over the last 50 ns and across all three replicates of each simulation system in the all-atom simulations, with the standard error of the mean of the interaction energy being given in an error bar.

Figure 6A–D show the protein–lipid binding energies of hIAPP oligomers with lipids of different types. In the AA simulation, both nonbonded Lennard-Jones and Coulomb binding energies, and their sum were calculated. Coulomb or electrostatic energy accounted for 50% or more of the total interaction energies of DPPC, DLPC, POPS, and GM1. However, Coulomb energy was less than 20% of the total energy of CHOL. Overall, the interaction energy increased with the increase in the size of the oligomers. Regarding the CO-raft, protein–lipid interaction energies followed the ranking order of DLPC > DPPC > CHOL. Regarding the PS-raft, the ranking order was POPS > DLPC ~ DPPC > CHOL. Regarding the GM-raft, the order was GM1 >> DLPC ~ DPPC > CHOL. Interestingly, the protein–GM1 interaction energy was greater than the protein–POPS interaction energy for each oligomeric size.

Similar patterns of protein–lipid interaction energy at CG resolution were also observed (Figure SD1). Since protein–lipid interaction energy depends on the number of lipids surrounding the membrane-bound protein, the normalized interaction energy, defined as the interaction energy divided by the number of lipids within the energy sampling range of 1.2 nm, was calculated at both CG and AA resolution, and the results are shown in Figure SD2. A similar pattern of interaction energy ranking order was also evident. 

In addition to the energy ranking order, we also directly compared the interaction energies between protein and lipid in both CG and AA simulations (Figure SD3). We discovered that the protein–lipid interaction energies of CHOL, DPPC and DLPC in the AA simulations were mostly higher than those in the CG simulations. In contrast, the protein–lipid interaction energy of GM1 in the AA simulation was lower than that in the CG simulation. These results suggest that the protein had stronger interactions with non-GM1 lipids but weaker interactions with GM1 lipids in the AA simulation when compared with the results of the CG simulation. The difference may be associated with the intrinsic differences between the CG and AA force fields. 

Finally, the inter-chain protein–protein binding energies of the membrane-bound oligomers involving two chains in the dimer and four chains in the tetramer were also examined at AA resolution (Figure 6E). Stronger interaction energy among chains was observed in the tetramer than in the dimer. Regarding the dimer, the binding energy was similar for all raft membrane types. However, regarding the tetramer, the interaction energy for the CO-raft, ~−2200 kJ/mol, was significantly greater than that for the PS-raft, ~1400 kJ/mol, and GM-raft, −1100 kJ/mol, which is indicative of a strong protein–protein association of the tetramer on the CO-raft. The protein–protein interaction energy of the membrane-bound oligomers was also examined at CG resolution (Figure SD4). Although a similar trend was observed for the dimer, no significant differences in the binding energy of the tetramer on different raft membranes were observed at CG resolution when compared with the results at AA resolution.

### 2.5. Membrane Disruption Behavior of [hIAPP]_n_

**Figure 7:**
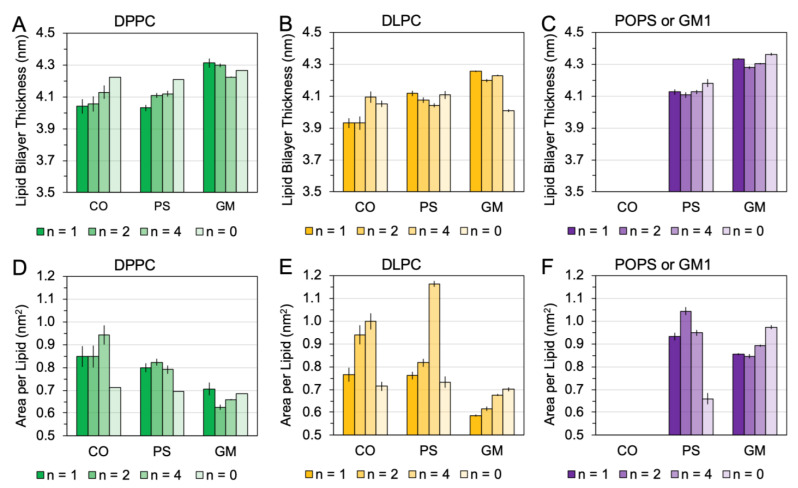
Bilayer thickness and area-per-lipid surface area of the 0.5 nm annular lipids surrounding the membrane-bound hIAPP aggregates bound to the raft membranes. The calculated bilayer thickness (**A**–**C**) and area per lipid (**D**–**F**) of annular lipids of different types, DPPC (**A**,**D**), DLPC (**B**,**E**), and POPS or GM1 (**C**,**F**), surrounding the membrane-bound hIAPP aggregates of different sizes, *n* = 1 (monomer), 2 (dimer), and 4 (tetramer), with the atoms of lipids and protein within 0.5 nm are shown. The bilayer thickness and area per lipid of the lipids in the absence of the protein (*n* = 0) are also shown as controls. Each data point represents the time average over the last 50 ns of the all-atom simulations and across all three replicates of each simulation system. The standard error of the mean is also shown as the error for each data point.

**Figure 8:**
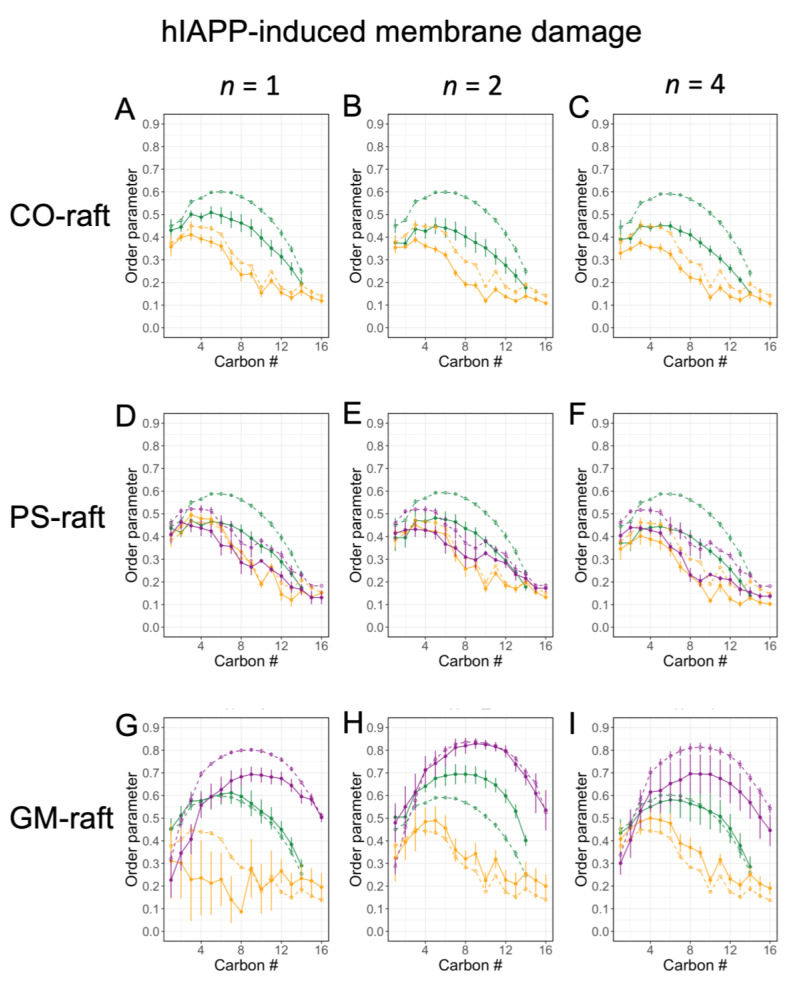
Lipid acyl chain orientational order parameter of the 0.5 nm annular lipids surrounding the membrane-bound hIAPP aggregates on the raft membranes. Profiles of lipid order parameters (solid lines) of annular lipids within 0.5 nm of hIAPP aggregates in different raft membranes are shown. Plots of lipid acyl chain orientational order parameter (filled symbols) vs. acyl chain number of the annular lipids of different types, DPPC (green), DLPC (orange), and POPS or GM1 (purple), with the atoms of lipids and protein within 0.5 nm surrounding the membrane-bound hIAPP aggregates of different sizes, *n* = 1 or monomer (**A**,**D**,**G**), 2 or dimer (**B**,**E**,**H**) and 4 or tetramer (**C**,**F**,**I**), on CO-raft (**A**–**C**), PS-raft (**D**–**F**), and GM-raft (**G**–**I**) are shown. As controls (dotted lines), the order parameters of lipids outside the 0.5 nm annular lipid shell or non-annular lipids (see Section 4) are shown in open symbols in each plot. Each data point represents time and replicate average over the last 50 ns and across all three replicates from both acyl chains of each diacyl lipid type. The error bar indicates standard error of the mean of each data point.

To investigate [hIAPP]_n_-induced membrane disruption, three physical parameters of lipids in the 0.5 nm annular lipid shells of raft membranes, i.e., *bilayer thickness*, *area per lipid* (*APL*), and *lipid acyl chain orientational order* (*lipid order parameter*), were calculated, and the results are shown in Figure 7 and Figure 8. The time and replicate averages of these physical parameters over the last 50 ns and across all three simulation replicates were determined using the AA simulation data (see Section 4).

Figure 7 shows the averaged bilayer thickness and APL of different lipid types, DPPC, DLPC, POPS, and GM1, in the 0.5 nm annular lipid shell for all three raft membranes. As controls, the averaged bilayer thickness and APL of the lipids in the raft membranes in the absence of the protein (*n* = 0) are also presented. 

Regarding DPPC (Figure 7A,D), a general trend of a decrease in bilayer thickness and a concomitant increase in APL in the presence of the protein was evident. The observed protein-induced effects appeared to be independent of the size of the oligomer. In addition, the protein-induced increase in APL was much stronger in the CO-raft than in the PS-raft, e.g., an APL value of 0.95 nm^2^ in the CO-raft vs. that of 0.80 nm^2^ in the PS-raft with the hIAPP tetramer, as shown in Figure 7D. Comparatively, no significant changes were found in bilayer thickness due to the protein in the GM-raft. However, a small “dip” in the APL value at 0.62 nm^2^ was evident when compared with the APL value of ~0.70 nm^2^ of the control in the GM-raft (Figure 7D). 

Regarding DLPC (Figure 7B,E), an overall trend of a decrease in bilayer thickness and a concomitant increase in APL in the presence of the protein was also found, except bilayer thickness in the PS-raft, where no significant change in bilayer thickness due to the protein was evident. Interestingly, we observed a much larger effect of protein-induced increase in APL in the PS-raft than in the CO-raft, e.g., an APL value of 1.15 nm^2^ in the PS-raft vs. 1.0 nm^2^ in the CO-raft with the hIAPP tetramer, as shown in Figure 7E. An opposite trend was found in the GM-raft. Here, a large increase in bilayer thickness and a decrease in APL due to the protein were evident. Here, bilayer thickness of ~4.2 nm and APL of 0.6 nm^2^ in the presence of the hIAPP monomer vs. 4.0 nm and 0.7 nm^2^, respectively, in the controls were observed. 

Regarding POPS (Figure 7C,F), specific to the PS-raft, the protein slightly decreased bilayer thickness but strongly increased APL, similar to the observation regarding DPPC or DLPC above. Regarding GM1 (Figure 7C,F), specific to the GM-raft, the protein had no strong effects on bilayer thickness, slightly decreasing bilayer thickness, but significantly decreased APL. The above protein-induced effects appeared to be independent of the size of the oligomer. 

As a third physical parameter of lipids to assess membrane disruption, Figure 8 shows the order parameter of different lipid types, DPPC, DLPC, POPS, and GM1, in the 0.5 nm annular lipid shell for all three raft membranes. Here, the [hIAPP]_n_-induced changes in the order parameter vs. carbon number of the lipid acyl chain, or transverse lipid order profile (see Figure 1), were systematically examined. Specifically, the order parameter profiles of lipids in the 0.5 nm annular lipid shells (filled symbols) were directly compared with those of the lipids outside the 0.5 nm annular lipid shells (open symbols), or non-annular lipids, for all lipid types. 

In the CO-raft and PS-raft (Figure 8A–F), a general trend of decrease in the order parameter profile of all lipid types due to the protein was evident. Here, a large decrease in the order parameter of DPPC from ~0.6 to ~0.45 near the middle of the acyl chain due to the protein was found, and the protein-induced change appeared to be independent of the size of the hIAPP oligomer. In contrast, a smaller decrease, less than 0.1, in the order parameter of DLPC or POPS was observed, but the decrease was more prominent with the tetramer than with smaller oligomers. 

In the GM-raft, a very different effect of the protein on the order parameter profile was evident when compared with that relative to the CO-raft or PS-raft. The major effects of the protein were detected only with the hIAPP monomer and dimer. Here, with the hIAPP monomer (Figure 8G), a large decrease in the order parameter profiles of GM1 and DLPC was evident at small carbon numbers or near the headgroup region of each of those lipids. For example, at the 6th carbon position, the order parameter dropped from ~0.8 to ~0.6 and from ~0.45 to ~0.20 for GM1 and DLPC, respectively, due the presence of the monomer. With the hIAPP dimer, a large increase, instead of a decrease, in the DPPC order parameter was detected. For example, the DPPC order parameter increased from ~0.6 to ~0.7 near the 6th carbon position in the presence of the hIAPP dimer. Within the uncertainties of the calculations, no significant effects of the tetramer on the order parameter of any lipid due to the presence of the tetramer (Figure 8I) were detected.

In addition to the order parameters in the nearest 0.5 nm annular shells, the order parameters in other larger annular shells were also examined in the CO-raft, PS-raft, and GM-raft, as shown in Figures SE1–SE3, respectively. Collectively, the differences in the order parameters in a given annular shell vs. those in the non-annular lipids progressively diminished as the size of the annular shell increased from 0.5 nm to 2–3 nm. 

### 2.6. Surface-Induced Protein Folding of [hIAPP]_n_

**Figure 9:**
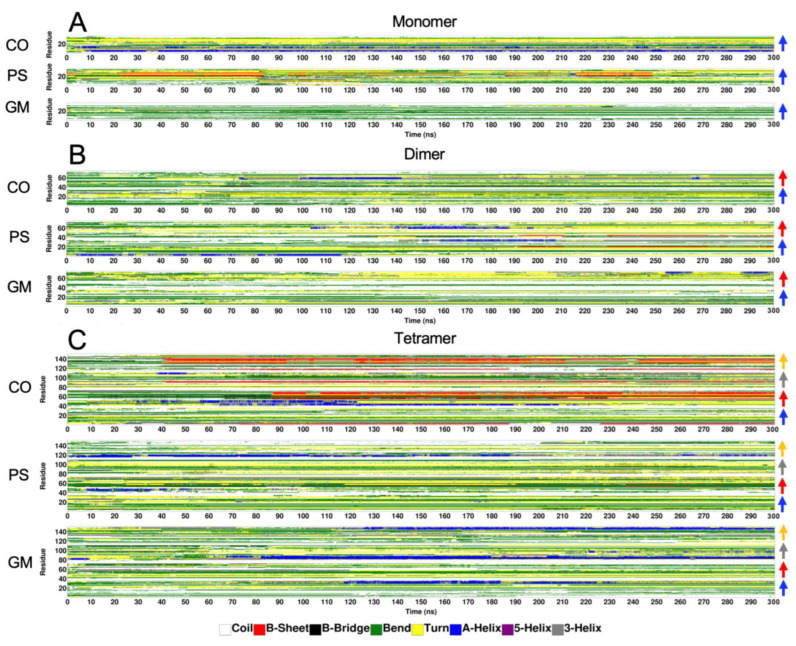
Time evolution of residue-resolved protein secondary structures of membrane-bound hIAPP on raft membranes. Representative plots of 3D color-coded protein secondary structures as functions of residue number (vertical axis) and simulation time (horizontal axis) in DSSP format (see Section 4) of membrane-bound hIAPP aggregates of different sizes, i.e., monomer (**A**), dimer (**B**), and tetramer (**C**), on CO-raft, PS-raft, and GM-raft are shown. The locations of the chains along the vertical axis are labeled with colored arrows, i.e., chain A (blue), chain B (red), chain C (gray), and chain D (orange), on the right side. The secondary structures are color-coded, i.e., coil (white), beta-sheet or B-sheet (red), beta-bridge or B-bridge (black), bend (green), turn (yellow), and alpha-helix or A-helix (blue), 5-helix (purple), and 3-helix (gray).

The time evolution and residue-resolved protein secondary structures of membrane-bound [hIAPP]_n_ bound to three raft membranes were determined using the DSSP algorithm (see Section 4), and the results of representative replicates for the monomer (Figure 9A), dimer (Figure 9B), and tetramer (Figure 9C) oligomers on the CO-raft (upper panel), PS-raft (middle panel), and GM-raft (lower panel) are demonstrated in Figure 9. Within the entire 300 ns AA simulations, evidence of surface-induced protein folding from non-hydrogen-bonded, disordered structures—such as bend (green) or coil (white)—to hydrogen-bonded, ordered structures—such as turn (yellow), helical (A-, 5-, or 3-helix, in blue, magenta, or gray, respectively), or planar beta (sheet and bridge, in red or black, respectively)—was found in different regions of the protein. DSSP plots of all 27 simulation replicates are shown in Figures SF1–SF3. Note that there were significant differences in the expression of secondary structures among the simulation replicates, indicating the highly complex protein folding landscapes of membrane-bound, intrinsically disordered protein on raft surfaces. A significantly large number of beta-sheet structures (red) were evident for the tetramer on the CO-raft. Interestingly, both highly stable intra-chain and inter-chain beta-sheets were evident, and these beta-sheets are clearly demonstrated in Figure 5C.

**Figure 10:**
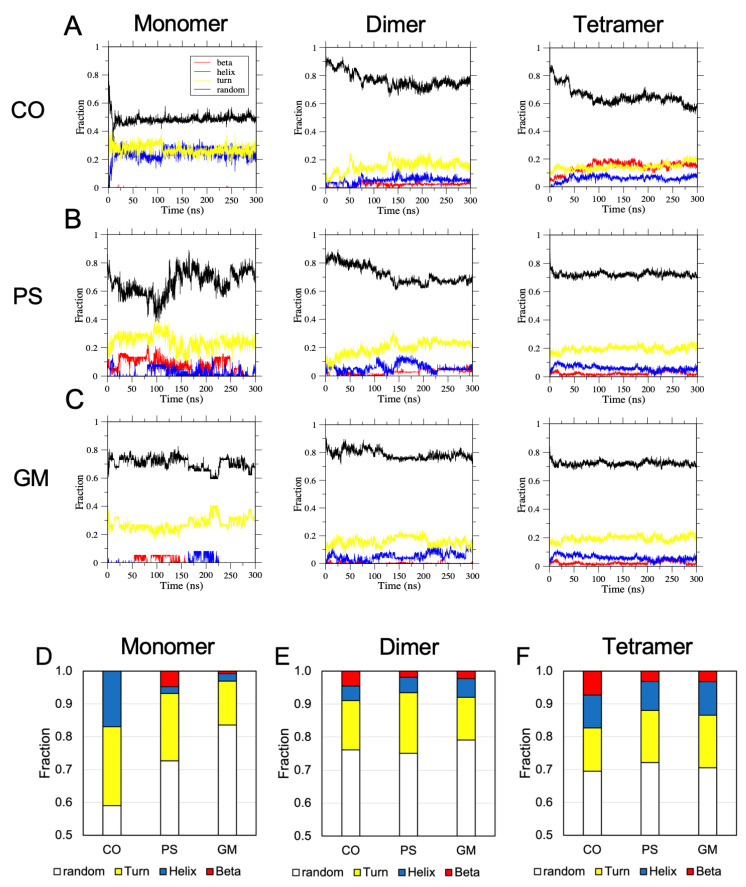
Protein folding kinetics and combined secondary structures of membrane-bound hIAPP aggregates on raft membranes. The fractions of residues that participated in four combined or re-classified secondary structures (see Section 4), i.e., beta (red), helix (blue), turn (yellow), and random (black), vs. time for membrane-bound hIAPP of different sizes, i.e., monomer, dimer, and tetramer, on the CO-raft (**A**), PS-raft (**B**), and GM-raft (**C**) are presented. In addition, we show the fractions of the combined secondary structures of the monomeric (**D**), dimeric (**E**), and tetrameric (**F**) membrane-bound hIAPP over the entire 300 ns in which each combined secondary structure was present over three independent replicates for each system. The error bar represents the standard error of the mean.

To better understand the protein folding kinetics, the eight secondary structures were re-grouped into four major structural categories, i.e., beta (sheet and bridge) in red, helical (helices of all sizes) in blue, turn in yellow, and random (bend and coil) in black. The fractions of these structural categories vs. simulation time, or protein folding kinetic plots, for the representative replicates in Figure 9 were calculated, and the results are shown in Figure 10A–C. In these representative folding kinetic plots, we observe that the alpha structure was consistently found in hIAPP oligomers of all sizes and on all raft surfaces. Yet, beta structures were also identified in oligomers of various sizes and on all raft surfaces, except in the monomer on the CO-raft surface (Figure 10A). The protein folding kinetic plots of all 27 replicates are shown in Figures SG1–SG3. The alpha structure was observed in 26 replicates, except in one replicate of the monomer on the PS-raft (Figure SG2C). In addition, no beta-sheet structure was evident in two replicates of the monomer on the CO-raft; only sporadic and small beta structures were detected in only one replicate (Figure SG1B). Not all protein folding kinetics of hIAPP oligomers exhibited equilibrated behaviors, particularly monomers on all raft surfaces, indicating that some membrane-bound protein complexes did not reach equilibrated structures within our 300 ns AA simulation time period.

In addition to individual protein folding kinetic plots, the number of occurrences of different secondary structures of all residues within the entire 300 ns AA simulation was determined for each replicate. The average fraction of each secondary structure over three independent replicates for each oligomer on three different raft membrane types is given in Figure 10D,E. Overall, regarding the hIAPP monomer, the average fraction of random conformations was the smallest on the CO-raft surface, with a value of ~60%, and the largest on the GM-raft surface, with a value of ~85%. In addition, the hIAPP monomer had a very small average fraction of beta conformations on both the CO-raft and the GM-raft. Regarding the dimer and tetramer, significant average fractions of helical and beta conformations were observed on all raft surfaces. There were no significant differences in the average fractions of secondary structures of hIAPP tetramers among the various raft surfaces.

## 3. Discussion

Using a multiscale molecular dynamics approach, the *physio-chemical behaviors of lipid binding, membrane disruption, and surface-induced folding* of hIAPP oligomers on nanostructured raft membranes were successfully characterized. The microsecond-long CG simulations of independent replicates allowed us to effectively sample the rotational and translational phase space of highly disordered oligomers in solution and on the planar membrane surfaces containing lipid nanodomains with and without PS- and GM1-clusters. Physiologically relevant information on lipid binding kinetics, residue-resolved binding patterns, and protein–lipid and protein–protein interaction energies on lipid nanodomains were obtained. The subsequent AA relaxation of the equilibrated, membrane-bound hIAPP aggregates following CG simulations further revealed the membrane disruption and folding behaviors of hIAPP oligomers on lipid nanodomains. These molecular events provide useful physio-chemical insights into the pathophysiological pathways of membrane damage mechanisms and the toxic structures of the early misfolded hIAPP aggregates that target the beta cells of the pancreas and other cell types, such as neurons, leading to type 2 diabetes and neurodegenerative diseases, respectively. 

The *binding preference* of disordered hIAPP oligomers for different lipid nanostructures was systematically examined. In this study, highly heterogeneous and dynamic lipid nanostructures, i.e., Lo, Ld, Lod, PS-clusters, and GM1-clusters, were created to model the nanodomain architecture of the outer and inner leaflet of the plasma membrane of a cell. Here, Lo, Ld, and Lod are non-specific nanodomains found on both leaflets, while PS-clusters and GM1-clusters are nanodomains exclusively found on the inner leaflet and outer leaflet, respectively. These transversely asymmetric distributions of nanodomains, therefore, provide different potential membrane targets for the attachment of misfolded hIAPP aggregates on cell membranes. As demonstrated in a recent study [16], in the absence of PS-clusters or GM1-clusters, disordered hIAPP oligomers of all sizes exclusively bind to the Lod domain of the CO-raft. However, in the presence of PS-clusters or GM1-clusters, these hIAPP oligomers exclusively bind to PS-clusters or GM1-clusters rather than to Lod domains. Here, the ranking order of hIAPP-raft binding time was CO-raft > PS-raft > GM-raft (see Table 1), and that of protein–lipid interaction energies was GM1 > POPS > DLPC > DPPC > CHOL (see Figure 6). Our results reveal that disordered hIAPP oligomers bind to the GM1 ganglioside in GM1-cluster nanostructures on the outer surface of plasma membranes much more strongly than other nanostructures, i.e., PS-cluster, Lo, Ld, and Lod nanodomains. 

Lipid molecules are asymmetrically distributed along the normal of the lipid bilayer in biological cell membranes [31]. Gangliosides and PS are exclusively located on the outer leaflet and the inner leaflet of plasma membranes, respectively. Although hIAPP is originally created inside beta cells and secreted to the extracellular space, recent studies have indicated that the damage to beta cells and other cell types, e.g., neurons, leading to cytotoxicity is mainly located on the outer leaflets of plasma membranes [1,28,29,33]. Since gangliosides are exclusively found on the outer leaflet of plasma membranes, our observation of the strong binding preference of hIAPP oligomers for GM1-clusters provides new computational evidence that hIAPP–ganglioside binding to the outside surface of plasma membranes is physio-chemically feasible. Note that several experimental cellular studies [28,29] on hIAPP–GM1 binding and the subsequent cytotoxicity of these hIAPP–GM1 complexes on neuronal cells have been demonstrated and thus validate our computational prediction. Note that the major ganglioside on the outer leaflet of beta cells is GM3, with a smaller carbohydrate headgroup than GM1 [1]. A recent simulation study further revealed strong hIAPP–GM3 binding on a model membrane [34]. It is important to mention that hIAPP oligomers also bind to anionic PS-clusters but with less interaction energy than anionic GM1-domains. Yet, the hIAPP–POPS interaction energy is still stronger than that of hIAPP–DLPC or –DPPC in Lod nanodomains. Therefore, in the absence of gangliosides, PS-clusters on the inner leaflet or Lod domains on both the inner and outer leaflets of cell membranes are alternative membrane binding domains for hIAPP oligomers. 

The *residue-resolved lipid binding pattern* of hIAPP oligomers binding to different lipid nanostructures was systemically investigated. Here, we discovered that the hydrophobic amino acids, L16 and I26, (see Figure 4 and Figure 5), are the major lipid binding residues of hIAPP oligomers on Lod and PS-cluster nanodomains on both the CO-raft and PS-raft. The *N*-terminal region (residues 1 to 20) containing residue L16 of structured hIAPP has been implicated in various experimental and computational studies [24,26,34,35,36]. In this study, we discovered that residue I26, associated with the *C* terminus (residues 21 to 31), which contains the fibril core of hIAPP fiber, represents an extra binding region for disordered oligomers. Therefore, our simulation study predicts that both residues are involved in the early membrane binding of disordered oligomers to *all leaflets* of plasma membranes. On the other hand, a rather interesting hIAPP–GM1 binding pattern (see Figure 4) was discovered. Regarding the monomer, all protein residues bind to GM1. Regarding the dimer, all protein residues, except the region around L16, bind to GM1. However, regarding the tetramer, only a fraction of the *N* terminus, residues 1–12, binds to GM1, and residues L16 and I26 are excluded from lipid binding. These observations lead us to conclude that in plasma membranes, specific hIAPP binding to nanodomains containing Lod (both leaflets) and PS-clusters (inner leaflet) mainly involves L16 and I26 of hIAPP via hydrophobic interactions. On the other hand, binding to GM1-clusters on the outer leaflet of plasma membranes is non-specific for the monomer but excludes either one or both of residues L16 and I26 for larger-size oligomers. The protein–lipid interactions between hIAPP oligomers and GM1-clusters are mainly hydrophilic interactions. These lipid binding patterns provide new physio-chemical insights for future drug intervention, particularly anti-aggregation therapy, aiming at inhibiting hIAPP aggregate attachment or the growth of hIAPP on different leaflets of plasma membranes. 

*Protein-induced membrane damage* is an important cytotoxic mechanism of amyloidogenic protein [1,5]. A reduction in bilayer thickness, an increase in APL, and a decrease in the lipid order parameter in the annular lipid shell surrounding the membrane-bound oligomers were evident in the annular lipid shell on both the CO-raft and PS-raft. These observations suggest that hIAPP oligomers perturb the biophysical properties of lipids on both leaflets and the PS lipid on the inner leaflet of plasma membranes. In contrast, in the GM-raft, an *increase* in the bilayer thickness of DLPC but a *decrease* in the APL of both DLPC and GM1 were detected, indicating that a different membrane damage mechanism occurred on the outer leaflet of the plasma membrane where GM1 was found. Interestingly, the decrease in the GM1 lipid order due to the monomer and the increase in the DPPC lipid order due to the dimer, but no significant change in the lipid order of all lipid types due to the tetramer, were evident. These observations suggest that small oligomers of hIAPP disrupt the membrane structures more effectively than large oligomers in the GM-raft. 

*Surface-induced protein folding* from disordered (coil and bend) to ordered (alpha helices, beta-sheets, and turn) structures was evident upon protein binding to the lipid domains of our raft membranes. Previous experimental and simulation studies indicate that alpha helices represent the initial folded structures upon hIAPP binding to membranes and that the subsequent formation of beta-sheets further leads to major membrane damage and fibril growth [26,35,37]. In this study, we observed significant alpha-helical formation in membrane-bound monomers on the CO-raft but not on the PS-raft or GM-raft. Therefore, the Lod domain promotes alpha-helical folding upon protein binding, while anionic PS- or GM1-clusters do not. However, the PS-clusters on the PS-raft promoted beta-sheet formation more strongly than the Lod domain and GM1-clusters on the PS-raft and GM-raft, respectively, with hIAPP monomers.

As the size of oligomers increased from monomer to dimer to tetramer, beta-sheets were identified on all raft membranes. Our results, therefore, lead us to conclude that the Lod domains on both leaflets of plasma membranes only support alpha-helical attachment for monomeric hIAPP. However, Lod domains support both alpha-helical and beta-sheet attachments for larger oligomers. The presence of both stable alpha and beta structures in the membrane-bound dimer and tetramer on all raft membranes suggests that both secondary structures may contribute to the formation of alpha-barrel pore and beta-barrel pore, as proposed in a recent study [38]. In addition, the prevalence of beta-sheet structures in larger-size oligomers on the surfaces of all three raft membranes suggests that beta-sheets exposed to solvent may act as seeds to recruit other amyloidogenic proteins to aggregate on the membrane surface or promote fibril growth. This surface-induced fibril growth has been implicated as a major membrane damage mechanism of hIAPP aggregates and other membrane-active amyloid aggregates [2,33,39,40]. Since Lod domains are found on both leaflets of plasma membranes, this surface-induced beta-sheet aggregation may operate or occur both inside and outside the beta cells of the pancreas and other non-pancreatic cells, such as neurons. 

## 4. Materials and Methods

### 4.1. Raft Membranes and hIAPP Oligomers

Three phase-separated raft membranes, CO-raft, PS-raft, and GM-raft, at CG resolution were used in this study. The CO-raft is a single lipid bilayer of saturated and unsaturated phosphatidylcholines (PCs) and cholesterol (CHOL) in water. It contains 828 saturated dipalmitoyl-PC (DPPC), 540 unsaturated dilinoleoyl-PC (DLPC), 576 CHOL, and 66,741 water molecules, in a lipid molar ratio of DPPC:DLPC:CHOL = 0.42:0.28:0.30. In the PS-raft or the GM-raft, some lipids on one lipid leaflet are replaced with 1-palmitoyl-2-oleoyl-PS (POPS) or monosialotetrahexosylganglioside (GM1), respectively. The molecule count of the asymmetric PS-raft is 36 GM1, 709 DPPC, 407 DLPC, 410 CHOL, and 56,114 water molecules, in a lipid molar ratio of POPS:DPPC:DLPC:CHOL = 0.08:0.24:0.28:0.30. The molecular count of the asymmetric GM-raft is 162 POPS, 666 DPPC, 540 DLPC, 576 CHOL, and 65,365 water molecules, in a lipid molar ratio of GM1:DPPC:DLPCLCHOL = 0.02:0.43:0.20:0.25. Each raft membrane has a size of ~22 × 22 × 20 nm^3^. Under the physiological conditions of 0.1 M NaCl, 310 K, and 1-atmosphere pressure, the CO-raft contains highly dynamic, ordered DPPC-rich and CHOL-rich (Lo) domains, disordered DLPC-rich (Ld) domains, and mixed DPPC-DLPC (Lod) domains for up to 20 μs in CG simulations. Regarding the PS-raft or GM-raft, PS-clusters within the Lod domain or GM1-clusters within the Lo domain on one lipid leaflet are observed, respectively. Details on the construction, energy minimization, position-restraining equilibration, and unconstrained CG MD simulation of protein membranes in the NPT ensemble based on Martini CG forcefields [41] and ran on the GROMACS-4.6.7 MD simulation program [42] can be found elsewhere [16,17,18,30]. All simulations were carried out under the physiological conditions of 0.1 M NaCl, 310 K, and 1-atmosphere pressure as described above. 

hIAPP oligomers at CG resolution in solution were created using a self-aggregation process from monomers. To create an hIAPP monomer, a 16-residue-long random-coil hIAPP_1–16_ was attached to a single 21-residue-long peptide hIAPP1_7–37_ extracted from an atomistic (AA) Cryo-EM structure [15]. After the AA-to-CG resolution transformation [43], followed by a 5 μs CG simulation, a disordered monomer in solution was created. To generate an hIAPP dimer or tetramer in solution, two or four monomers were separately lined up along the x-direction or x-y-directions using a replication tool [42], followed by a 5 μs CG simulation. All hIAPP oligomers or [hIAPP]_n_—monomer (*n* = 1), dimer (*n* = 2), and tetramer (*n* = 4)—in solution were highly dynamic and sampled extensive conformational space based on protein residue-contact map analysis [44]. All CG simulations were performed under the physiological conditions of 0.1 M NaCl, 310 K, and 1-atmosphere pressure. The procedures and conditions of MD simulations of CG [hIAPP]_n_ in solution identical to those of raft membranes in solution are described above [16,17,18]. All construction tools were based on the GROMACS-MD simulation program package and are described in detail in our previous studies [16,17,18].

The primary sequence of hIAPP is **K**CNTATCATQ**R**LANFLVHSSNNFGAILSSTNVGSNTY. To characterize the hydrophobicity profile of the primary sequence of the full-length hIAPP monomer, a hydropathy index vs. residue number plot [45] of the 37-residue-long peptide hIAPP1 was created [16]. Based on a 5-point moving average fit to the hydrophobicity profile, two major hydrophobic peaks at residue #16 (L16) and residue #26 (I26), with hydropathy indices >2.0, were identified and are highlighted in red in the above primary sequence. In addition, hIAPP carries a net charge of +2e, with the two positively charged amino acids being at residue #1 (K1) and residue #11 (R11), which are highlighted in bold in the above primary sequence. These four key amino acids allowed us to interpret the residue-resolved oligomer binding results based on the known hydrophobic and charge properties of the peptide. 

### 4.2. Multiscale Simulations of Oligomer Binding to Raft Membranes

Placing the pre-equilibrated hIAPP oligomer at a distance above the raft membrane surface represents the starting simulation structure of our oligomer–raft molecular complex. In this study, three independent simulation replicates, replicate 1, replicate 2, and graph replicate 3, were separately created for each oligomer–raft complex. Here, replicate 1 was placed above the center of the surface of the lipid leaflet (upper leaflet), with the minimum distance between any atom of the protein and any atom of the lipid >5 nm. Replicates 2 and 3 were subsequently created with the protein position shifted by +2 nm and −2 nm along the x-direction relative to the protein position of replicate 1, respectively. The use of three independent simulation replications for each oligomer–raft complex allowed us to perform the efficient phase sampling of protein–membrane binding events. Upon generating the initial structures, the same CG MD simulation procedures for oligomers or raft membranes in solution were performed for up to 15 μs or longer. A few replicates were extended to 20 μs to ensure that at least 5 μs long, stable, and equilibrated protein–membrane attachment was achieved in the protein–membrane binding events. A total of 27 CG simulation systems involving oligomers of 3 aggregation sizes (*n* =1, 2, and 4), three raft membranes, and 3 simulation replicates for each oligomer–raft complex with an accumulated CG simulation time of over 400 μs were generated. Since the CG simulation time based on the Martini force field is about four times faster than the real time based on the CG diffusion rate of water [30], this study modeled, simulated, and predicted more than 1.5 milliseconds of biological processes associated with the early aggregation of disordered hIAPP oligomers on membrane surfaces with different nanostructures.

After the CG simulations, each of the CG oligomer–raft systems was converted into an AA structure using a CG-to-AA resolution transformation procedure [42]. The subsequent MD transformed AA structure was equilibrated with similar energy minimization and position-restraining procedures as in the simulations of the CG oligomer–raft complexes. However, instead of Martini CG force fields, the atomistic AMBER14SB [46] force field for proteins and the SLIPIDS [47,48] force field for lipids were used in all AA MD simulations. 

### 4.3. Classifications of Lipid Nanodomains and Annular Lipids

Three major phase-separated nanodomains (DPPC-rich, liquid-order domain (Lo); DLPC-rich, liquid disordered domain (Ld); and mixed Lo/Ld domain with mixed DPPC and DLPC (Lod)) were classified using a data-filtering tool, g_select, by GROMACS [42]. The classification of lipids into Lo, Ld, and Lod domains was based on the proximity threshold of 0.5 nm between any two atoms of DPPC and DLPC, as described in detail elsewhere [16,17]. All phospholipids in the CO-raft were classified into Lo-DPPC, Ld-DPPC, Lod-DPPC, and Lod-DLPC. Finally, Lo-CHOL, Ld-CHOL, and Lod-CHOL represent groups of CHOL molecules, for which at least one CHOL atom is within 0.5 nm of the PC lipid atoms in the Lo, Ld, and Lod domains, respectively [17]. Regarding the PS-raft, POPS mainly partitioned in the Lod domains and formed PS-clusters on one leaflet. Regarding the GM-raft, GM1 exclusively partitioned in the Lo domains and also formed GM1-clusters on one leaflet. Hence, our raft membranes provided five different lipid nanostructures, Lo, Ld, Lod, PS-cluster, and GM-cluster domains, that provided the membrane binding targets for hIAPP oligomers. 

The same g_select tool was also used to characterize annular lipid (AL) shells from each oligomer–raft complex upon protein binding. If an atom of any lipid was within a certain threshold range, e.g., 0.5 or 1.2 nm, from an atom of an oligomer, that lipid was assigned to the 0.5 nm or 1.2 nm AL shell, accordingly. In addition, AL shells, each with two thresholds, e.g., 1–2 nm, representing lipids with any of their atoms >1 nm but <2 nm from any atoms of the protein were also classified. In this study, five AL shells with thresholds of 0.5, 0.5–1, 1–2, 2–3, and 1.2 nm were generated. 

The time- and replicate-averaged number of lipids in each of the 5 lipid domains (Lo, Ld, Lod, PS-cluster, and GM1-cluster) or AL shell over the last 5 μs of CG simulation and 50 ns of AA simulation were calculated to assess the domain or annular shell composition of our raft systems upon protein binding. As controls, the 5 lipid domains in the absence of protein in the simulations were also determined to assess the effects of membrane-bound oligomers on domain composition. Similarly, the composition of lipids outside the AL shells, or non-annular lipids (nALs), was also determined and compared with that of AL shells to assess the specificity of lipid types surrounding membrane-bound oligomers upon protein binding. 

### 4.4. Membrane Binding Behaviors of Oligomers

The qualitative visualization of the kinetics of hIAPP–membrane binding and the lipid types surrounding membrane-bound hIAPP oligomers for each replicate was performed using a molecular visualization program, VMD [49].

The quantitative analysis of the lipid binding kinetics and protein residue-resolved lipid-binding sites of hIAPP oligomers were performed using the minimum-distance analysis tool *mindist* by GROMACS [42]. Briefly, a plot of protein–lipid minimum distance (*mindist*), defined as the minimum distance between any protein atom and the atom of its binding lipid or water neighbors, vs. simulation time was used. In addition, the number of contacts of the mindist within an interaction threshold (2 nm) vs. simulation time was also determined. Finally, the time-averaged *mindist* vs. protein residue number over the last 5 μs of the 15 μs long CG simulation or the last 50 ns of the 300 ns long AA simulation was calculated. These three parameters, *mindist* vs. time (upper panel), number of contacts vs. time (mid panel), and mindist vs. residue number (bottom panel), are presented as a 3-panel plot of each replicate. The first two panels were used to investigate the kinetics of protein binding in terms of the time event of protein attachment, or lipid binding time, to each lipid type. The last plot provides important information about the mindist of the nearest-neighbor lipids or water surrounding the protein upon forming a membrane-bound state. This plot is also defined as the *mindist* spectrum in this study to quantify the residue-resolved lipid binding or water binding regions of each hIAPP oligomer interacting with the raft membrane. Time and replicate averages were determined for the *mindist* spectral analysis to evaluate the effect of the oligomer size on the binding behaviors of hIAPP oligomers.

Similarly, the time- and replicate-averaged nonbonded potential energies between oligomers and each lipid type, or protein–lipid interaction energy, were collected using the tool *energy* by GROMACS [42] for each oligomer–raft complex. Both Van Der Waals, or Lennard-Jones, and electrostatic, or Coulomb, potential energies of protein–protein and protein–lipid interactions were separately calculated in the CG and AA simulations of all oligomer–raft systems. Since the nonbonded energy sampling threshold is ~1.2 nm, the protein–lipid interaction energy was divided by the number of lipids in the 1.2 nm AL shell to determine the normalized protein–lipid interaction energy in both CG and AA simulations. Details on protein–lipid binding potential energy calculations are given elsewhere [16,17].

### 4.5. Characterization of Membrane Disruption by Oligomer Binding to Raft Membrane

To evaluate the spatially resolved membrane disruption, or membrane damage, of membrane-bound protein on raft membranes, three biophysical membrane structural parameters, i.e., lipid bilayer thickness, area per lipid (APL), and lipid chain orientational order, in each 0.5, 0.5–1, 1–2, or 2–3 nm AL shell, as well as in the corresponding nAL (as a control), were determined for each AA oligomer–raft complex. 

The bilayer thickness and APL of each PC lipid were calculated using an MD trajectory analysis program, Fast Analysis Toolbox for Simulations of Lipid Molecules, or FATSLiM [50]. The lipid orientational order parameter as a function of the carbon number of the lipid acyl chain was calculated using the *order* tool by GROMACS [42]. This lipid order parameter is a measurement of the tilt of three sequentially connected carbon atoms along the PC acyl chains with respect to the normal of the bilayer and thus provides a transverse (along the bilayer normal) profile of acyl chain ordering in the AL shells surrounding the membrane-bound protein [47,51]. The time- and replicate-averaged values of bilayer thickness, APL, and lipid order across the last 50 ns of the simulations and three independent replicates were systematically determined for each AA oligomer–raft complex.

### 4.6. Secondary Structures of Membrane-Bound Oligomers

The residue-resolved secondary structure of membrane-bound oligomers in every time step was calculated using the tool *do_dssp* by GROMACS [42] based on Define Secondary Structure of Proteins (DSSP) [52]. We classified the beta-sheet and beta-bridge structures into a single beta group, and the three helical structures, alpha-helix (A-helix), p-helix (or 5-helix), and 310 helix (3-helix), into a single helix group to simplify the analysis of surface-induced folding behaviors of hIAPP on raft membranes. The fraction of amino acid residues in each re-classified secondary structure vs. simulation time was employed to examine the protein folding kinetics of each membrane-bound oligomer.

## 5. Conclusions

In conclusion, our multiscale MD study provides new mechanistic insights into disordered hIAPP oligomer binding and protein folding behaviors on highly dynamic and heterogeneous nanostructures. We discovered that the hydrophobic residues (L16 and I26) are the major sites for the lipid binding of hIAPP oligomers to the Lod and PS-cluster domains and that the hydrophilic *N* terminus is the major region for the lipid binding of the tetramer to GM1-clusters. We discovered that GM1–hIAPP binding is the strongest among other lipid–hIAPP binding types, suggesting that hIAPP oligomers have a strong propensity to bind to the GM1 ganglioside of neurons other than to beta-cell membranes in the pancreas. Both the alpha-helix and the beta-sheet are stable secondary structures and were found on all nanodomains for large oligomers. These ordered structures may contribute to both ion pore formation and surface-induced fibril growth on both leaflets of plasma membranes. The surface-induced ordered structures on leaflet-specific lipid domains could provide useful insights for future drug intervention therapy targeting membrane-bound oligomers.

## Figures and Tables

**Table 1:** Summary of lipid binding time of CG-hIAPP oligomers from three independent simulation replicates and the replicate-averaged composition of 0.5 nm annular lipids surrounding the membrane-bound CG- and AA-hIAPP oligomers of different aggregation sizes, i.e., monomers (*n* = 1), dimers (*n* = 2), and tetramers (*n* = 4), on CO-raft, PS-raft, and GM-raft surfaces.

Simulation	*n*	Raft	Binding Time (μs)			CHOL% *	DPPC% *	DLPC% *	POPS% * or GM1% *	Number of Lipids *
CG	1	CO	3.84	8.41	0.26	17 ± 1	34 ± 2	49 ± 2		9.0 ± 0.2
		PS	1.19	0.91	0.29	20 ± 1	25 ± 2	27 ± 3	29 ± 3	10.3 ± 0.4
		GM	0.94	0.60	0.09	8 ± 4	8 ± 3	4 ± 1	80 ± 1	15.6 ± 0.8
	2	CO	7.85	0.61	4.03	19 ± 1	36 ± 3	45 ± 1		11.9 ± 0.6
		PS	2.99	0.19	0.70	22 ± 1	25 ± 2	23 ±1	30 ± 1	14.2 ± 0.3
		GM	0.40	0.16	1.50	4 ± 3	7 ± 3	6 ± 3	83 ± 16	16.7 ± 2.8
	4	CO	8.34	10.55	1.02	20 ± 2	34 ± 2	47 ± 3		16.3 ± 0.7
		PS	1.77	3.75	3.10	22 ± 1	22 ± 1	25 ± 1	31 ± 1	20.0 ± 0.7
		GM	0.44	0.96	1.20	4 ± 3	9 ± 3	6 ± 3	81± 8	24.2 ± 2.2
AA	1	CO				15 ± 4	27 ± 5	58 ± 9		18.2 ± 2.0
		PS				14 ± 2	36 ± 10	19 ± 12	30 ± 9	19.2 ± 3.3
		GM				17 ± 7	31 ± 10	12 ± 3	40 ± 7	15.7 ± 2.3
	2	CO				20 ± 10	32 ± 11	48 ± 14		23.6 ± 4.9
		PS				23 ± 5	28 ± 5	20 ± 9	29 ± 7	29.0 ± 6.5
		GM				4 ± 4	24 ± 7	26 ± 1	46 ± 5	15.0 ± 1.4
	4	CO				26 ± 3	29 ± 3	45 ± 5		31.4 ± 2.2
		PS				25 ± 5	24 ± 3	22 ± 7	30 ± 1	29.7 ± 1.0
		GM				11 ± 6	34 ± 3	18 ± 4	37 ± 1	22.6 ± 1.7

* The uncertainties are standard errors of the means over the last 5 μs or 50 ns of the CG or AA simulations across the three independent replicates, respectively.

## Data Availability

Not applicable.

## Supplementary Materials

The following supporting information can be downloaded at: https://www.mdpi.com/article/10.3390/molecules28104191/s1, Figures SA1–SA4: Lipid composition of the phase-separated lipid domains and annular lipids, Figure SB1: Protein–lipid and protein–water minimum distance spectra of membrane-bound hIAPP aggregates at all-atom resolution, Figures SC1–SC3: Structures of membrane-bound hIAPP aggregates in raft membranes, Figures SD1–SD4: Multiscale protein–lipid and protein–lipid interaction energies of membrane-bound hIAPP in raft membranes, Figures SE1–SE3: Membrane disruption profiles of annular lipid shells surrounding membrane-bound hIAPP aggregates in raft membranes, Figures SF1–SF3: Protein secondary structures of hIAPP aggregates on raft membranes in DSSP format, Figures SG1–SG3: Protein folding kinetics of hIAPP aggregates on raft membranes.

## Abbreviations

hIAPP	human Islet Amyloid Polypeptide
CG	coarse-grained
PC	phosphatidylcholine
PS	phosphatidylserine
GM	ganglioside
CHOL	cholesterol
DPPC	dipalmitoyl-PC
DLPC	dilinoleoyl-PC
Lo	liquid-ordered
Ld	liquid-disordered
Lod	mixed Lo/Ld
MD	molecular dynamics
AA	all-atom
AL	annular lipid
nAL	non-annular lipid
*mindist*	minimum distance
APL	area per lipid
POPS	1-palmitoyl-2-oleoyl-PS
