# Supplementary material for: Exploring the Role of Anionic Lipid Nanodomains in the Membrane Disruption and Protein Folding of Human Islet Amyloid Polypeptide Oligomers on Lipid Membrane Surfaces Using Multiscale Molecular Dynamics Simulations
Source: Molecules. 2023 May 19;28(10):4191. doi: 10.3390/molecules28104191 (PMC10223233; doi:10.3390/molecules28104191)
Supplement: Supplementary file 1 [file molecules-28-04191-s001.zip › molecules-2386654-supplementary.pdf]

molecules

MDPI

Article

Supplementary Information (SI) - Exploring the Role of Anionic Lipid Nanodomains in the Membrane Disruption and Protein Folding of Human Islet Amyloid Polypeptide Oligomers on Lipid Membrane Surfaces Using Multiscale Molecular Dynamics Simulations

Ngoc Nguyen [1], Amber Lewis [2], Thuong Pham [1], Donald Sikazwe [3] and Kwan H. Cheng [1,2,*]

[1] Physics Department., Trinity University, San Antonio, TX 78212, USA; nnguyen5@trinity.edu, tpham3@trinity.edu
[2] Neuroscience Department., Trinity University. San Antonio, TX 78212, USA; alewis2@trinity.edu
[3] Pharmaceutical Sciences Department, Feik School of Pharmacy, University of the Incarnate Word, San Antonio, TX 78209, USA; sikazwe@uiwtx.edu
* Correspondence: kcheng1@trinity.edu; Tel.: +1-210-999-8469

SI - A

The lipid compositions of the phase-separated lipid domains, Lo, Ld, Lod, and the annular lipids from the membrane-bound hIAPP aggregates, or $[hIAPP]_n$, of different sizes (n = 0, 1, 2, and 4) are shown in Figure SA1-1 and Figures SA2-3, respectively. The time variation of the number of annular lipids in the 0.5 nm annular lipid shell for the hIAPP tetramer in CO-raft is demonstrated in Figure SA2-4.

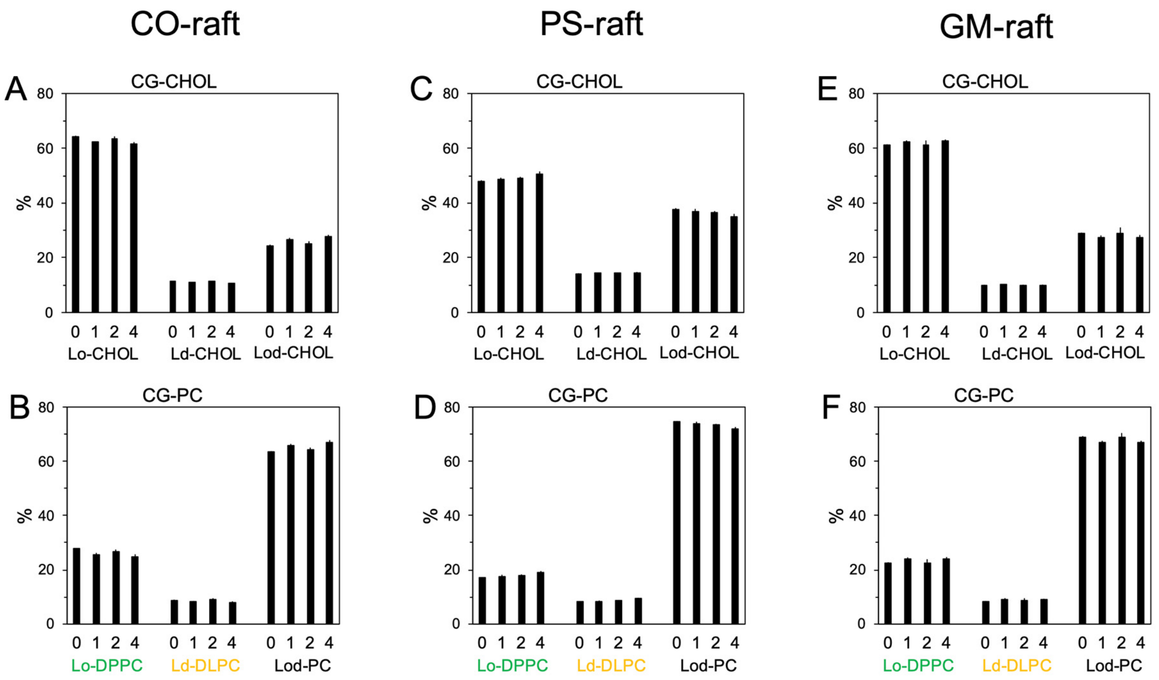

Figure SA1. Compositions of liquid-ordered (Lo), liquid-disordered (Ld), and interfacial Lo/Ld (Lod) in CO-raft, PS-raft, and GM-rafts at coarse-grained (CG) resolution. The time- and replicate-averaged percentages of CHOL, DPPC, DLPC, and GM1 in Lo, Ld, and Lod over the last 5 µs and across three independent simulation replicates of the CG simulations for the CO-raft (**A,B**) , PS-raft (**C,D**) and, GM-raft (**E,F**) in the presence of membrane-bound hIAPP aggregates of different sizes (n = 1, 2, and 4) are shown. The uncertainty, SE of mean, of each value is given. The compositions of raft membranes in the absence of protein (n = 0) are also presented as a control.

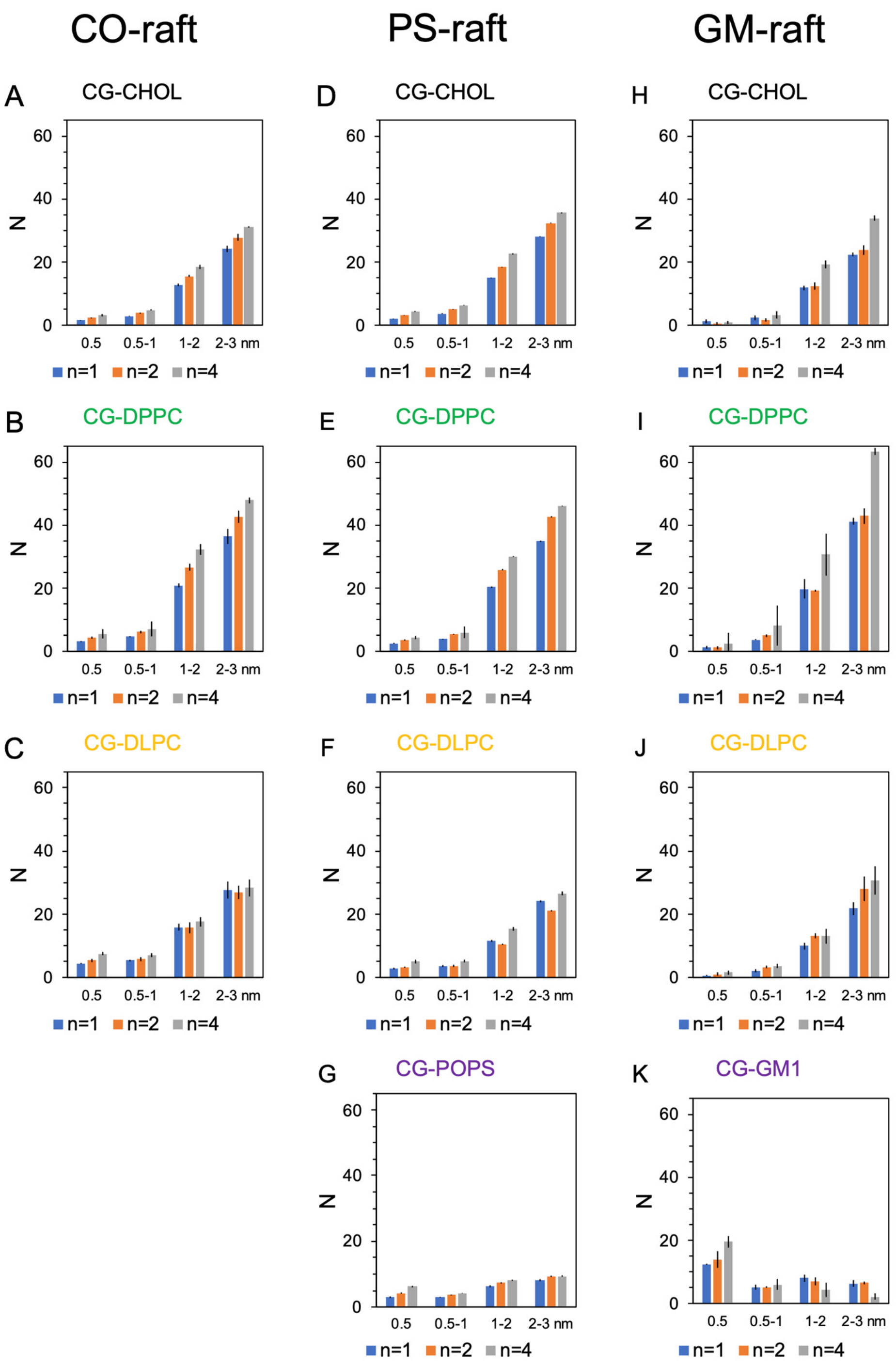

Figure SA2. Number of lipids in annular lipid shells. The time- and replicate-averaged number of lipids of different types, CHOL (**A**,**D**,**H**), DPPC (**B**,**E**,**I**), DLPC (**C**,**F**,**J**), POPS (**G**), and GM1 (**K**), in annular lipid shells of 0.5, 0.5-1, 1-2, and 2-3 nm away from the membrane-bound hIAPP aggregates of different sizes (n = 1,2, and 4) over the last 5 µs and across three independent simulation replicates of the CG simulations for CO-, PS-, and GM-rafts are shown. The uncertainty, SE of mean, of each value is given.

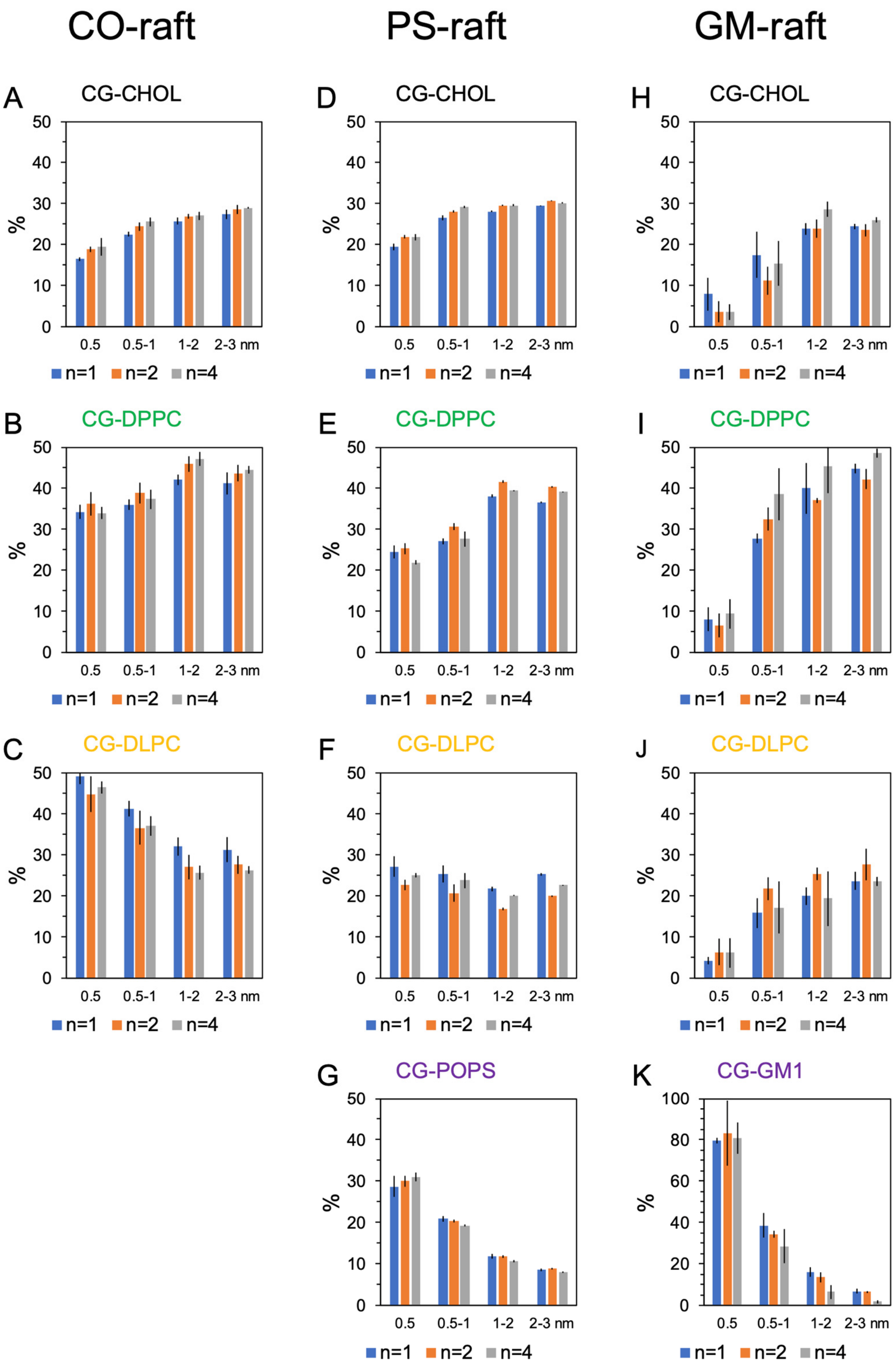

Figure SA3. Percentage of lipids in annular lipid shells. The time- and replicate-averaged percentage of lipids of different types, CHOL (**A**,**D**,**H**), DPPC (**B**,**E**,**I**), DLPC (**C**,**F**,**J**), POPS (**G**), and GM1 (**K**), in annular lipid shells of 0.5, 0.5-1, 1-2, and 2-3 nm away from the membrane-bound hIAPP aggregates of different sizes (n = 1, 2, and 4) over the last 5 µs and across three independent simulation replicates of the CG simulations for CO-, PS-, and GM-rafts are shown. The uncertainty, SE of mean, of each value is given.

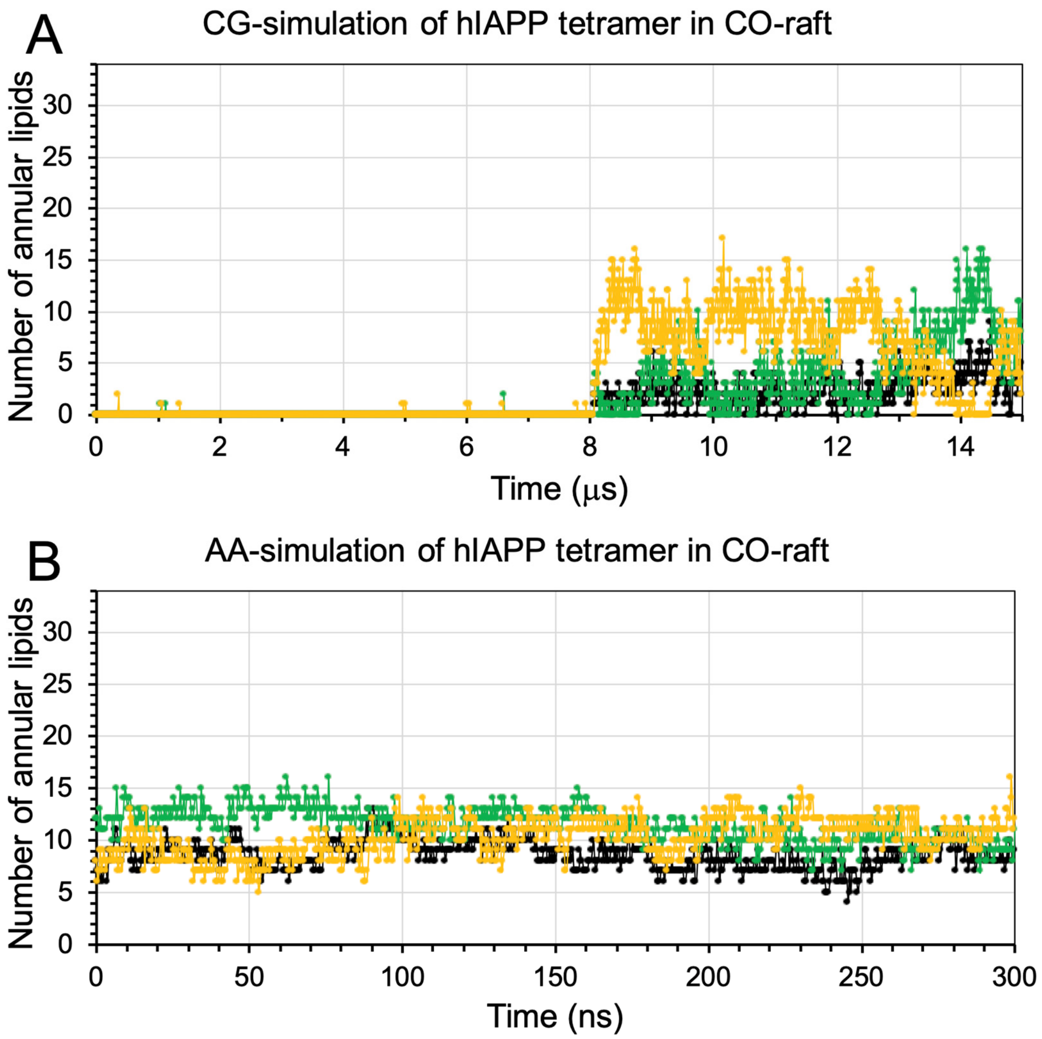

Figure SA4. Number of lipids in the 0.5 nm annular lipid shell vs. simulations time. The time variation of the number of lipids in the 0.5 nm annular lipids surrounding the hIAPP tetramer for a representative replicate at both CG (**A**) and AA (**B**) resolutions is demonstrated. The data for the lipids, DPPC (green), and DLPC (orange)are shown.

SI - B

Protein-lipid and protein-water minimum distance spectra of membrane-bound hIAPP aggregates of different sizes on CO-, PS-, and GM-rafts at all-atom resolution are shown in Figure SB1.

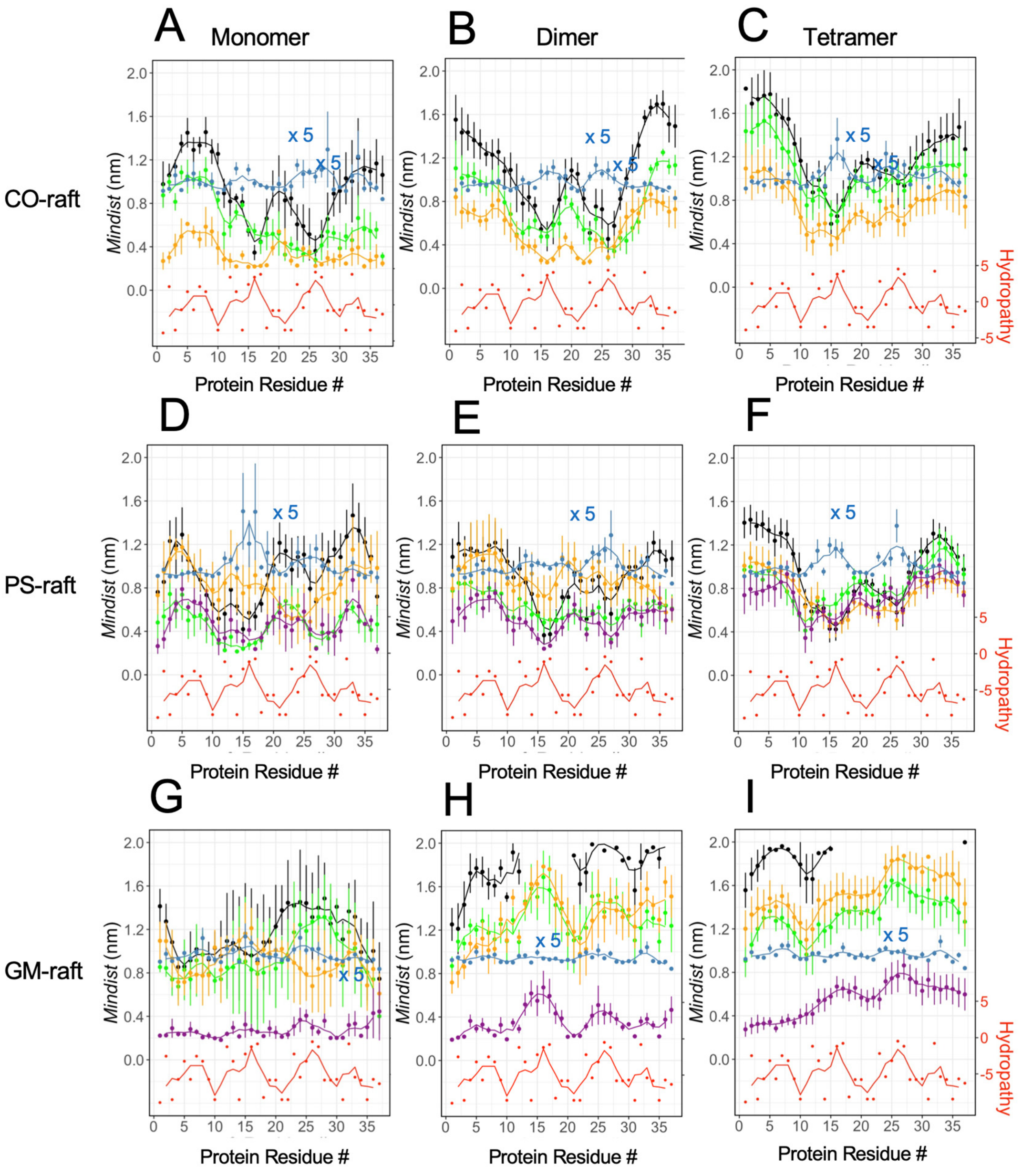

Figure SB1. Protein-lipid and protein-water minimum distance spectra of membrane-bound hIAPP aggregates of different sizes, monomer (**A,D,G**), dimer (**B,E,H**), and tetramer (**C,F,I**), on CO-raft (**A-C**), PS-raft (**D-F**), and GM-raft (**G-I**). The time-, protein chain, and replicate-averaged protein-lipid and protein-water minimum distance (*mindist*) vs. protein residue of membrane-bound hIAPP aggregates of various sizes over the last 50 ns, over all protein chains (chains A and B for dimer, and chains A-D), and across three independent simulation replicates of all-atom resolutions are shown. The data for the lipids, DPPC (green), DLPC (orange), CHOL (black), POPS (purple), GM1 (purple), and the water (blue) are shown. Due to the smaller mindist of protein-water, the values of mindist of water are magnified by 5 for clarity. In addition, the hydropathy vs. protein residue trace (red) for the hIAPP peptide is also plotted in each graph. The uncertainty, SE of mean, of each value is given.

SI - C

Structures of the membrane-bound hIAPP aggregates of different sizes (n = 1, 2, and 4) and the 0.5 nm annular lipid shells surrounding the proteins for the CO-raft (Figure SC1), PS-raft (Figure SC2), and GM-rafts (Figure SC3).

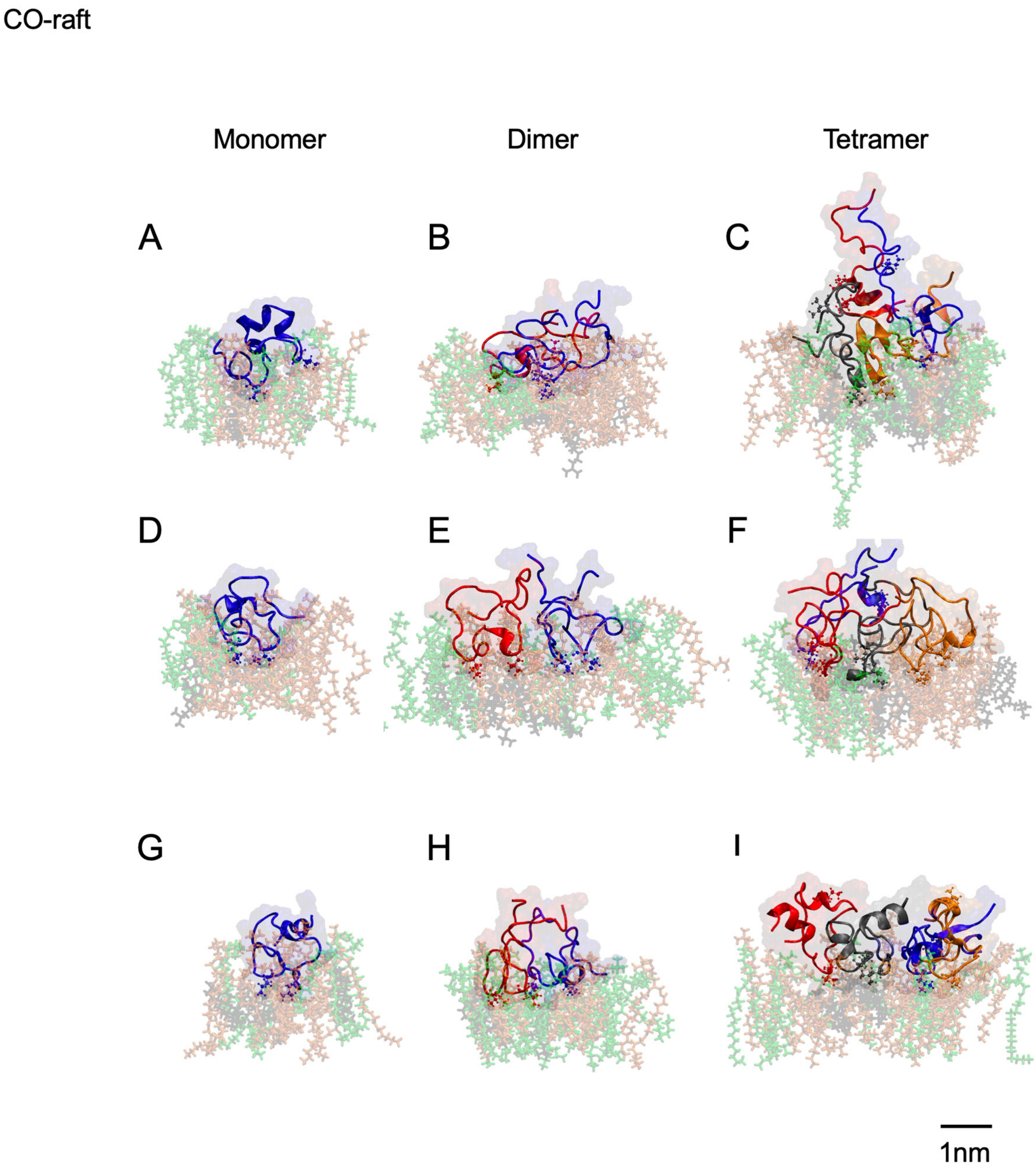

Figure SC1. Structures of membrane-bound hIAPP aggregates of sizes, n = 1 (monomer), n = 2 (dimer), and n = 4 (tetramer), from three independent replicates for each size, monomer (**A,D,G**), dimer (**B,E,H**), and tetramer (**D,F I**) in CO-raft. The protein chains are color coded, chain A (blue), chain B (red), chain C (gray), and chain D (orange), and their secondary structures are shown in ribbons. The two hydrophobic side chain residues, L16 and I26, of each chain are shown in licorice. The lipids, DPPC in green, DLPC in orange, and CHOL in black, within the 0.5 nm annular lipid shells for each hIAPP aggregate are also shown. A scale bar of 1 nm is also shown.

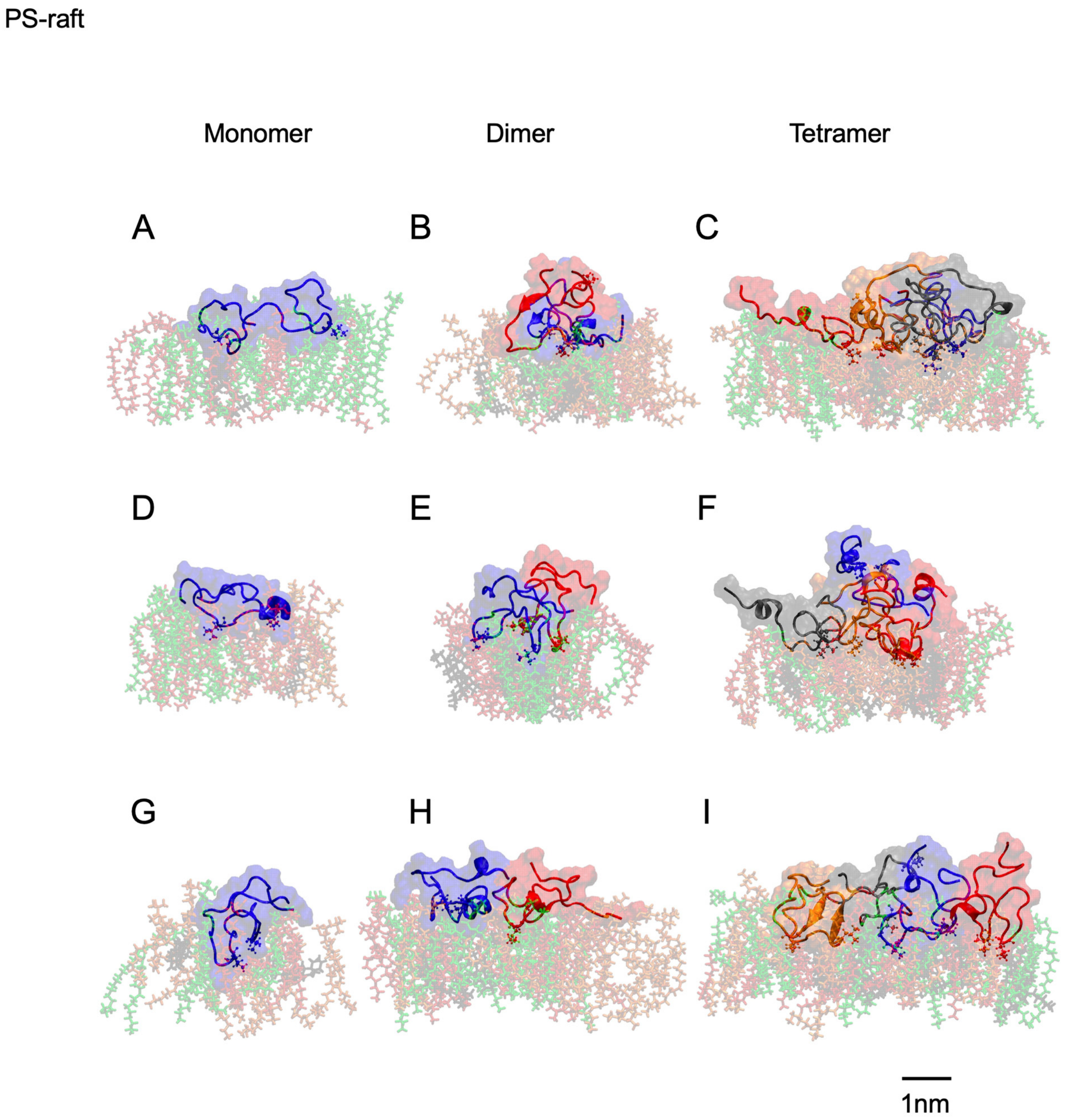

Figure SC2. Structures of membrane-bound hIAPP aggregates of sizes, $n = 1$ (monomer), $n = 2$ (dimer), and $n = 4$ (tetramer), from three independent replicates for each size, monomer (**A,D,G**), dimer (**B,E,H**), and tetramer (**D,F,I**) in PS-raft. The protein chains are color coded, chain A (blue), chain B (red), chain C (gray), and chain D (orange), and their secondary structures are shown in ribbons. The two hydrophobic side chain residues, L16 and I26, of each chain are shown in licorice. The lipids, DPPC in green, DLPC in orange, CHOL in black, and POPS in red, within the 0.5 nm annular lipid shells for each hIAPP aggregate are also shown. A scale bar of 1 nm is also shown.

GM-raft

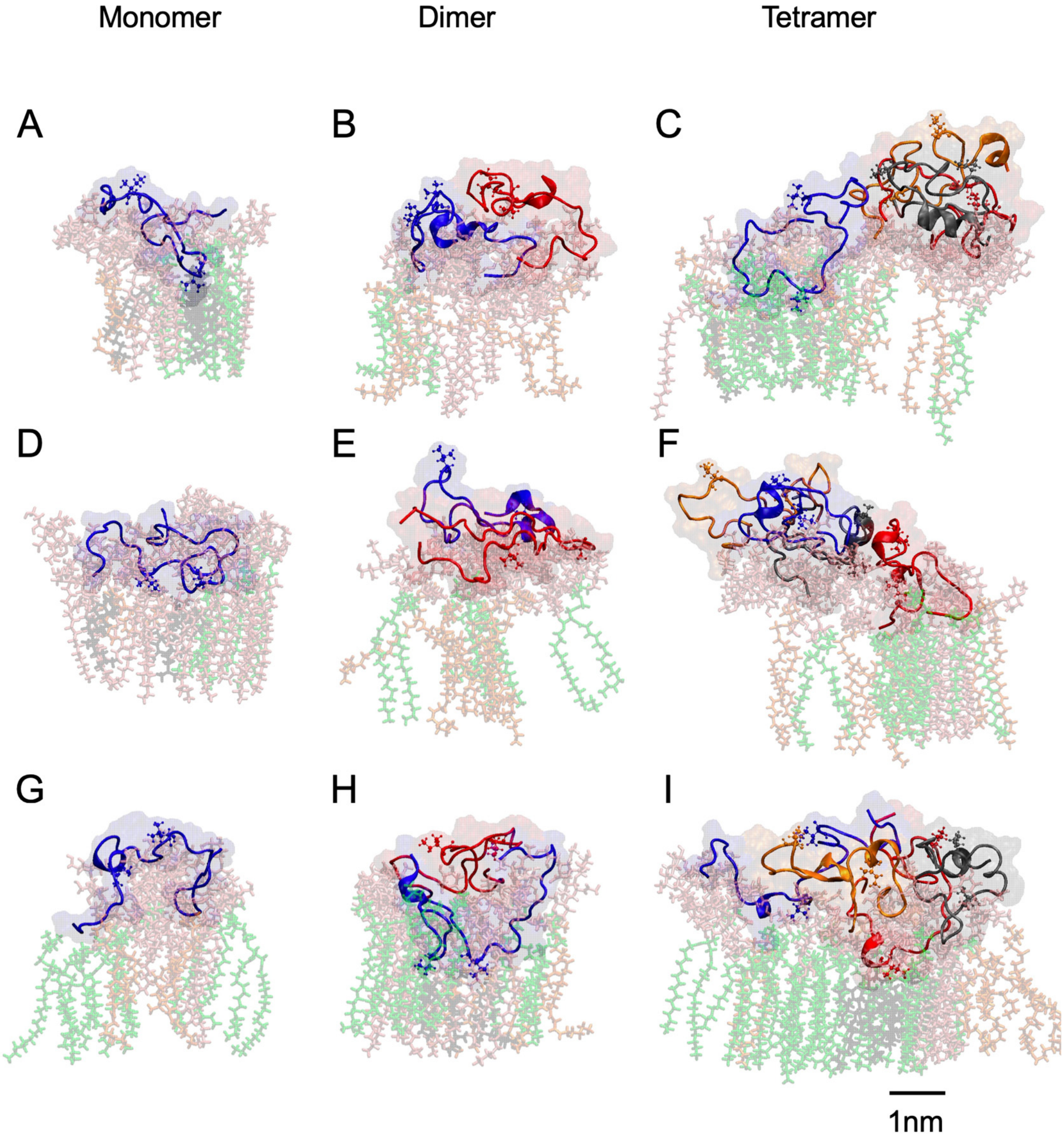

Figure SC3. Structures of membrane-bound hIAPP aggregates of sizes, $n = 1$ (monomer), $n = 2$ (dimer), and $n = 4$ (tetramer), from three independent replicates for each size, monomer (**A,D,G**), dimer (**B,E,H**), and tetramer (**D,F,I**) in GM-raft. The protein chains are color coded, chain A (blue), chain B (red), chain C (gray), and chain D (orange), and their secondary structures are shown in ribbons. The two hydrophobic side chain residues, L16 and I26, of each chain are shown in licorice. The lipids, DPPC in green, DLPC in orange, CHOL in black, and GM1 in pink, within the 0.5 nm annular lipid shells for each hIAPP aggregate are also shown. A scale bar of 1 nm is also shown.

SI - D

Multiscale total and normalized protein-lipid interaction energy (Figures SD1, SD2, and SD3) and protein-protein (Figure SD3) interaction energy of both coarse-grained (CG) and all-atom (AA) resolutions are shown.

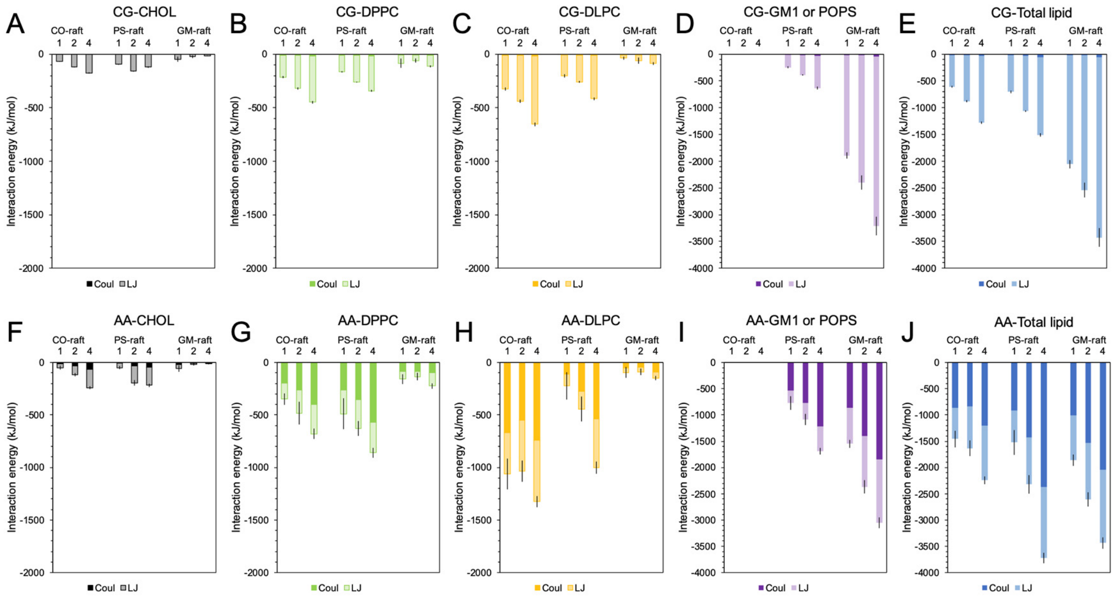

Figure SD1. Multiscale protein-lipid interaction energy of hIAPP aggregates in CO-, PS-, and GM-rafts. Total protein-lipid interaction energy (**A-J**) of different sizes (*n* = 1, 2, and 4) in CO-, PS-, and GM-rafts at coarse-grained (CG) resolution (**A-E**) and all-atom (AA) resolution (**F-J**) are shown. Results shown are for different lipid types, CHOL (black), DPPC (green), DLPC (orange), POPS or GM1 (purple), and total lipids (blue). The interaction energy involving electrostatic or coulomb (Coul) and van der Waals or Lennard Jones (LJ) are identified by darker and lighter colors, respectively. Each data point represents the time- and replicate-average over the last 5 μs in CG and 50 ns in AA. The error bar represents the standard error of the mean for the sum of Coul and LJ energies of each protein-lipid interaction energy value.

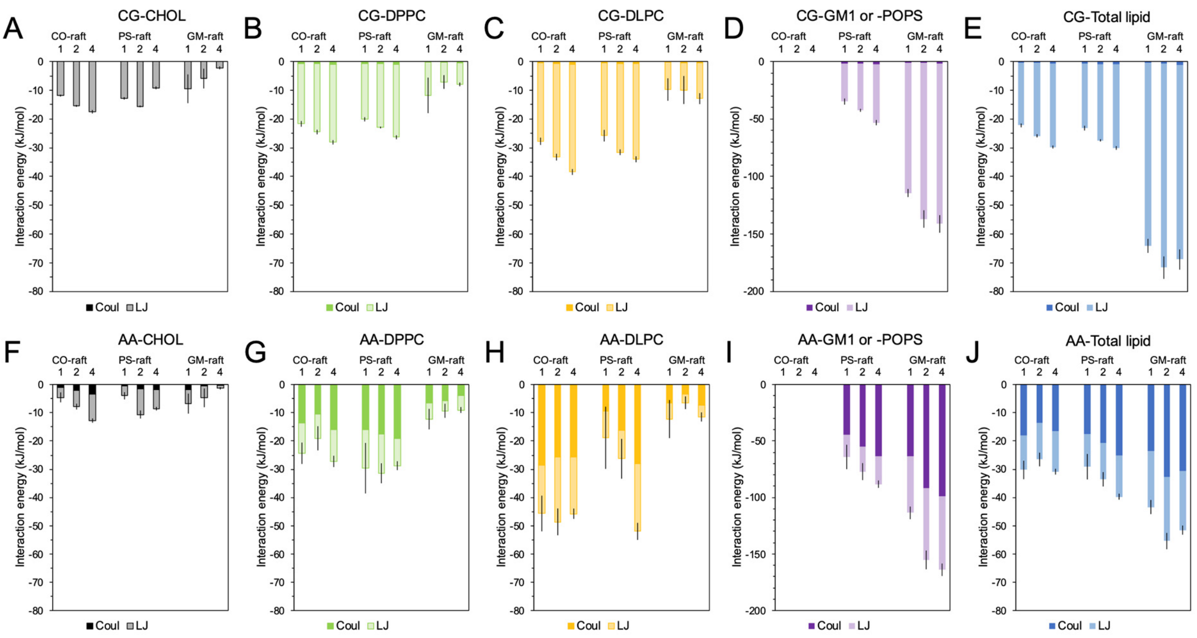

Figure SD2. Multiscale normalized protein-lipid interaction energy of hIAPP aggregates in CO-, PS-, and -GM-rafts. Normalized protein-lipid interaction energy (**A-J**) of different sizes (*n* = 1, 2, and 4) in CO-, PS-, and GM-rafts at coarse-grained (CG) resolution (**A-E**) and all-atom (AA) resolution (**F-J**) are shown. The normalized protein-lipid interaction energy is defined as the total protein-lipid interaction energy divided by the number of lipids within 1.2 nm from the protein, or energy sampling region (see Methods) for both CG and AA resolutions. See the legend of Figure SD1 for details.

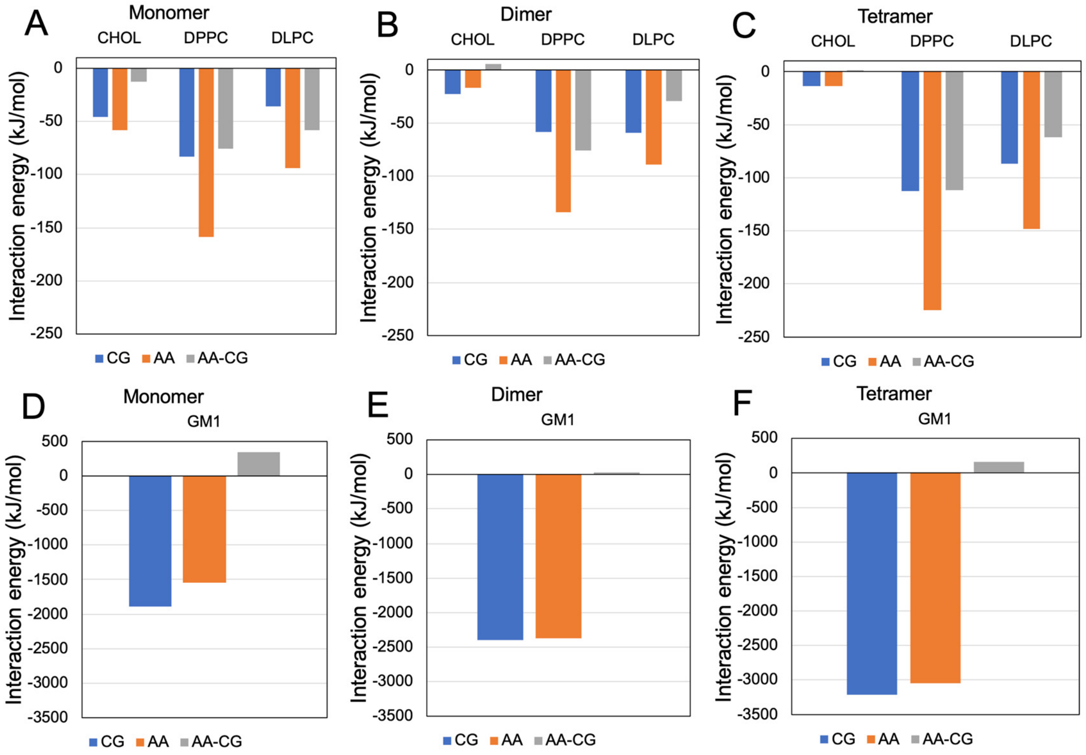

Figure SD3. Comparison of multiscale protein-lipid interaction energies of membrane-bound hIAPP aggregates in GM-raft. The total interaction energies between protein and lipid from CG (**A-C**) and AA (**D-F**) simulations for each lipid type and the differences are directly compared.

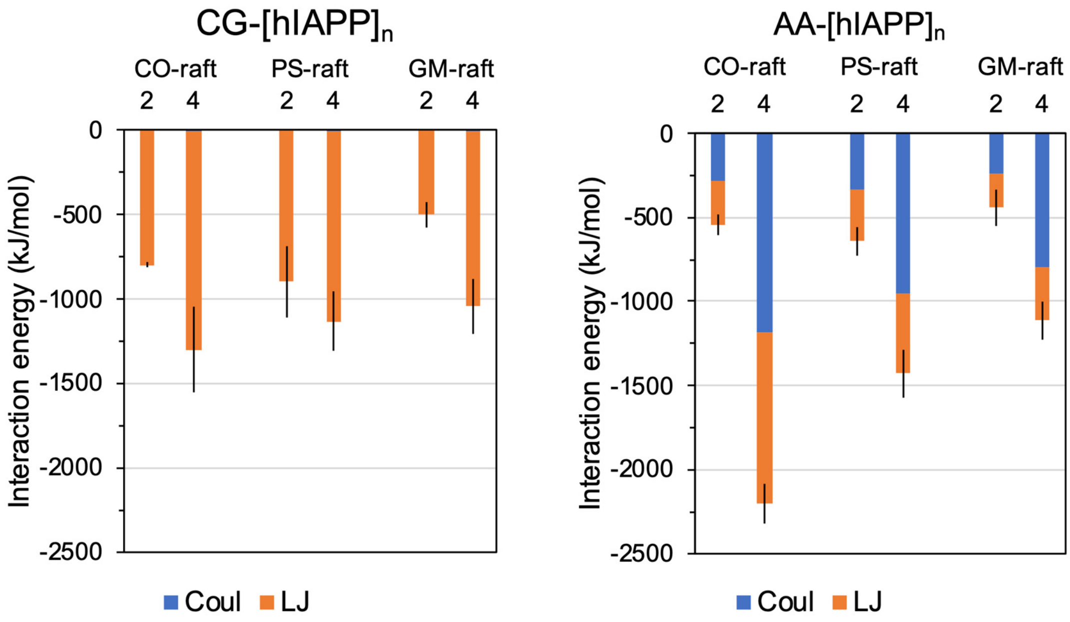

Figure SD4. Multiscale protein-protein interaction energy of membrane-bound hIAPP aggregates in CO-, PS-, and -GM-rafts. The interaction energy involving electrostatic or coulomb (Coul) and van der Waals or Lennard Jones (LJ) are identified by darker and lighter colors, respectively. Each data point represents the time- and replicate-average over the last 5 µs in CG and 50 ns in AA. The error bar represents the standard error of the mean for the sum of Coul and LJ energies of each protein-lipid interaction energy value.

SI - E

Membrane disruption profiles of hIAPP aggregates in CO-raft (Figure SE1), PS-raft (Figure SE2), and GM-raft (Figure SE3) based on lipid order parameter profiles in different annular lipid shells surrounding the protein.

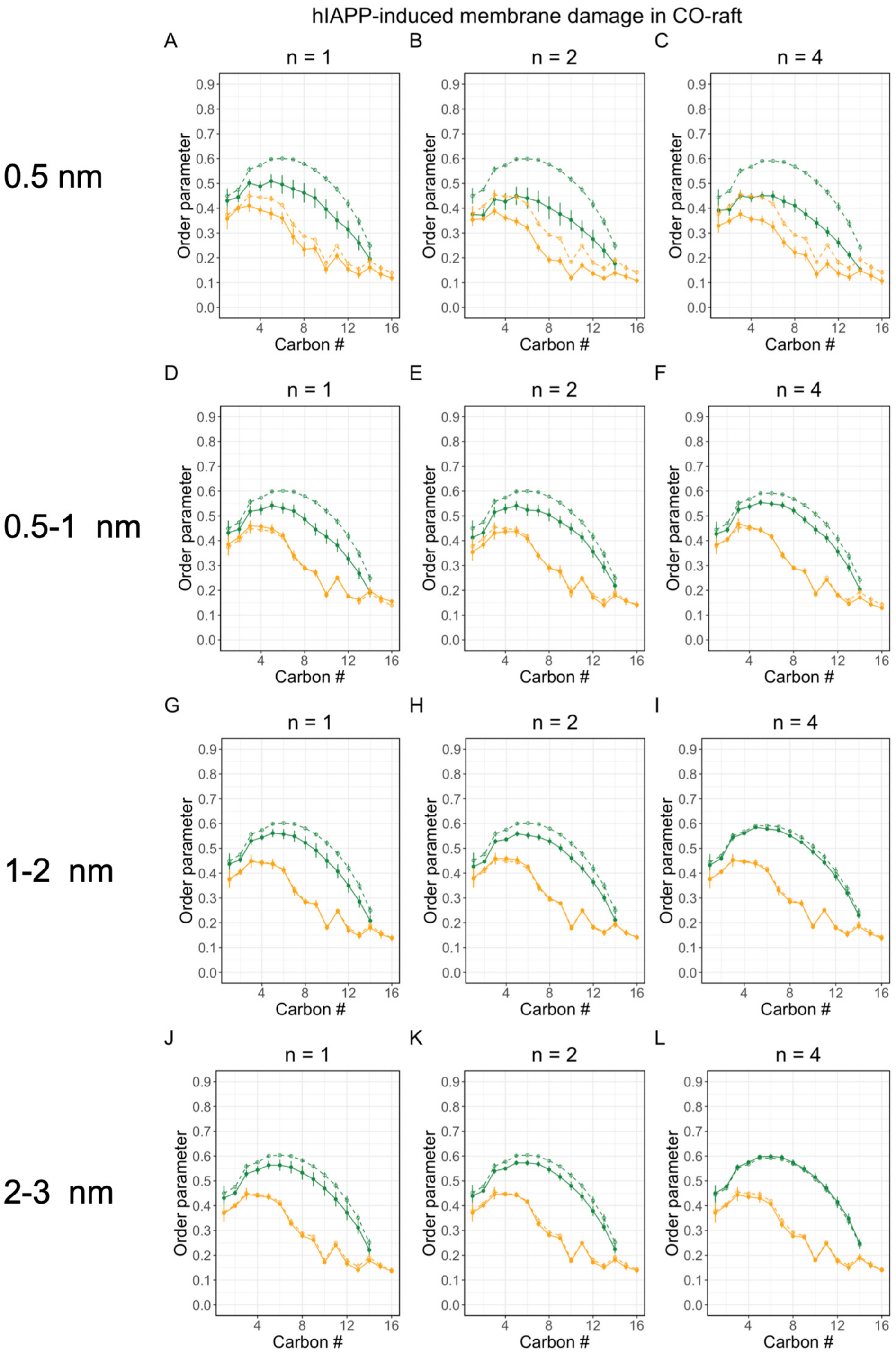

Figure SE1. Membrane disruption profiles of hIAPP aggregates sizes, n = 1 (monomer), n = 2, (dimer), and n = 4 (tetramer) in CO-raft. Lipid order parameters vs. chain carbon number of DPPC (green) and DLPC (orange) in the annular lipid (AL) shells (filled symbols) of 0.5 nm (**A-C**), 0.5-1 nm (**D-F**), 1-2 nm (**G-I**), and 2-3 nm (**J-L**) away from the protein as compared to those outside the AL shells (open symbols) are shown. Each data point represents the time- and replicate-average over the last 50 ns in AA. The error bar represents the standard error of the mean.

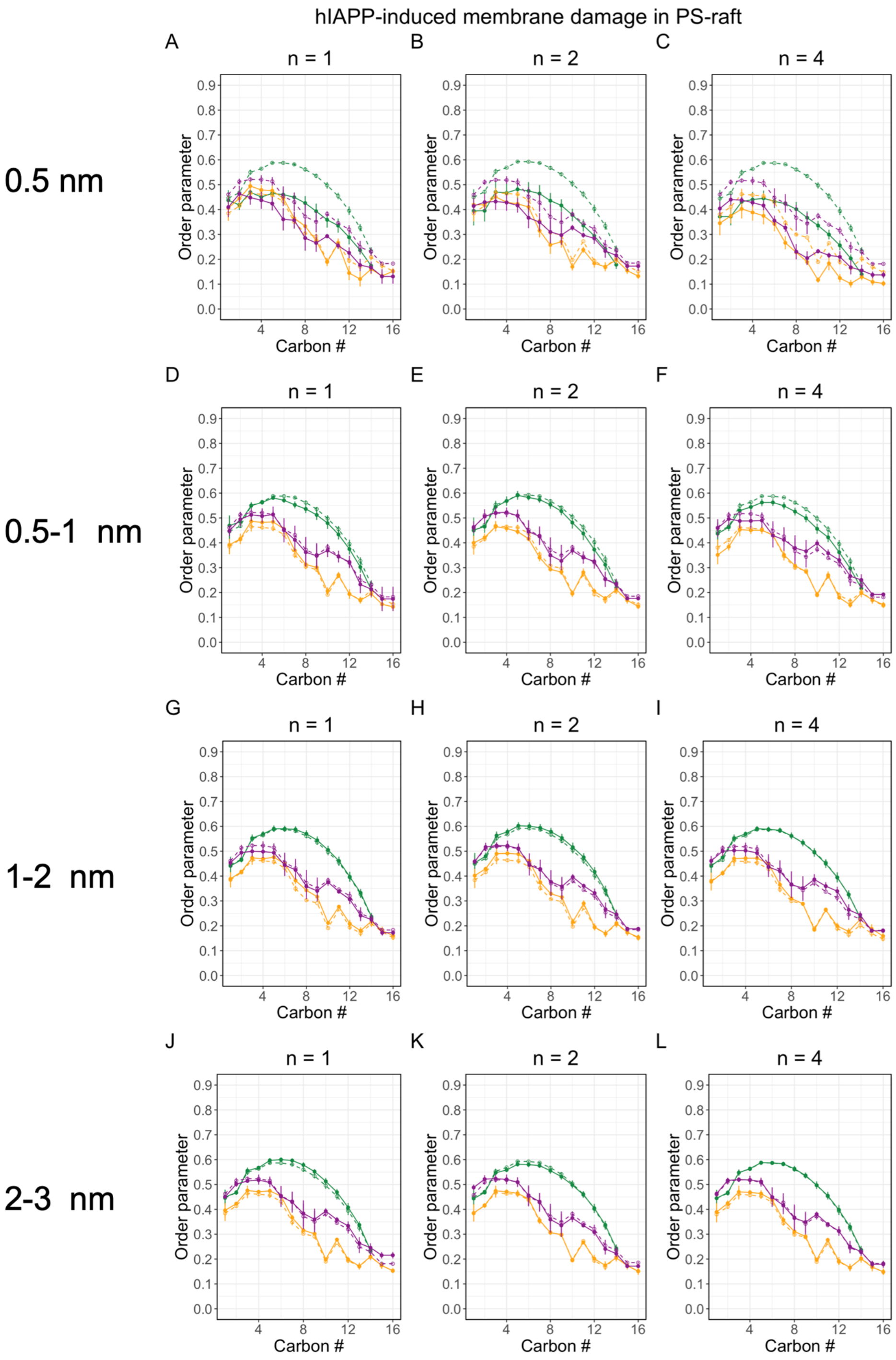

Figure SE2. Membrane disruption profiles of hIAPP aggregates sizes, n = 1 (monomer), n = 2, (dimer), and n = 4 (tetramer) in PS-raft. Lipid order parameters vs. chain carbon number of DPPC (green), DLPC (orange), and POPS (purple) in the annular lipid (AL) shells (filled symbols) of 0.5 nm (**A-C**), 0.5-1 nm (**D-F**), 1-2 nm (**G-I**), and 2-3 nm (**J-L**) away from the protein as compared to those outside the AL shells (open symbols) are shown. Each data point represents the time- and replicate-average over the last 50 ns in AA. The error bar represents the standard error of the mean.

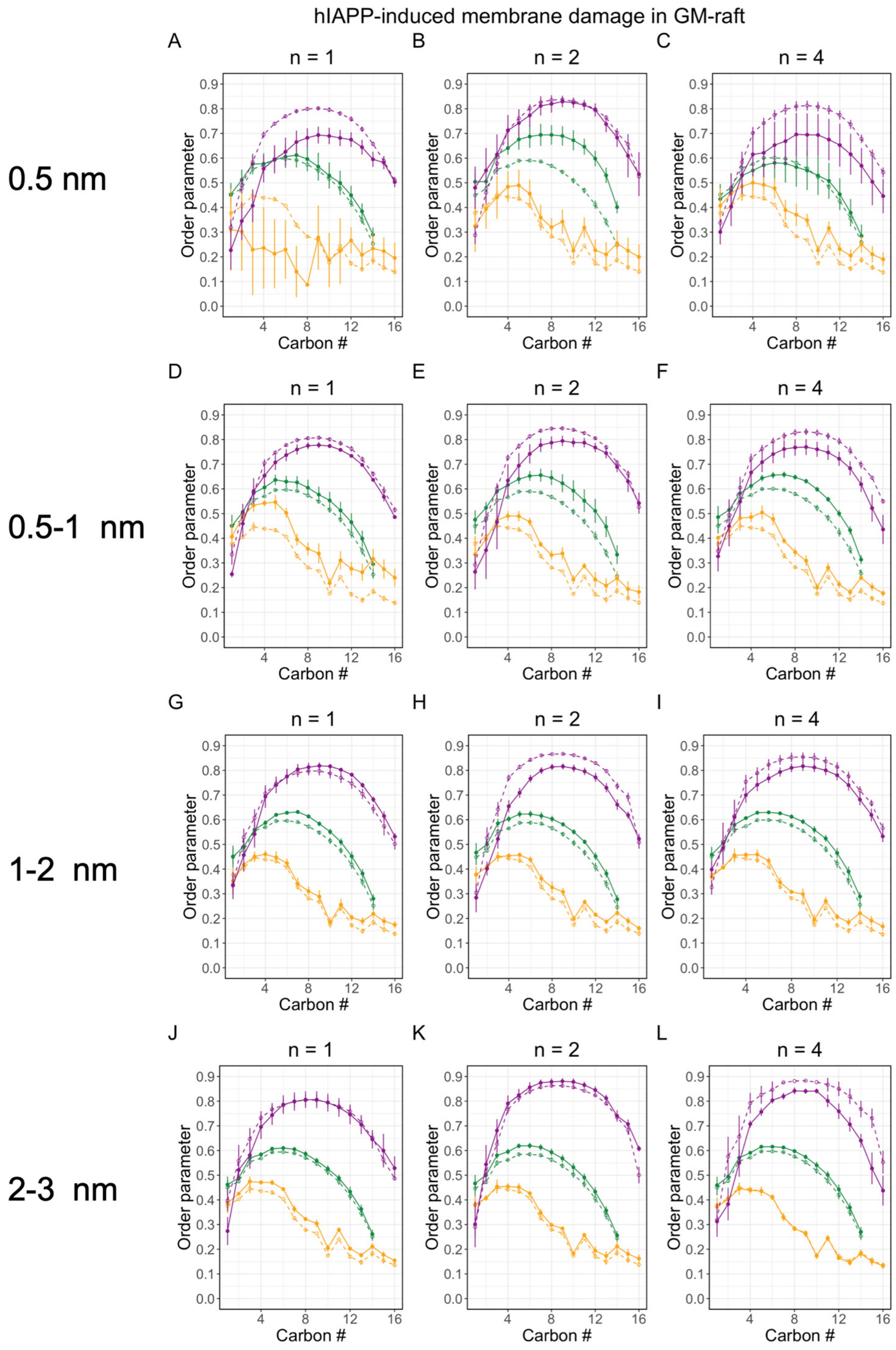

Figure SE3. Membrane disruption profiles of hIAPP aggregates sizes, n = 1 (monomer), n = 2, (dimer), and n = 4 (tetramer) in GM-raft. Lipid order parameters vs. chain carbon number of DPPC (green), DLPC (orange), and GM1 (purple) in the annular lipid (AL) shells (filled symbols) of 0.5 nm (**A-C**), 0.5-1 nm (**D-F**), 1-2 nm (**G-I**), and 2-3 nm (**J-L**) away from the protein as compared to those outside the AL shells (open symbols) are shown. Each data point represents the time- and replicate-average over the last 50 ns in AA. The error bar represents the standard error of the mean.

SI - F

Time-evolution of protein secondary structure of hIAPP aggregates upon binding to CO-raft (Figure SF1), PS-raft (Figure SF2), and GM-raft (Figure SF3).

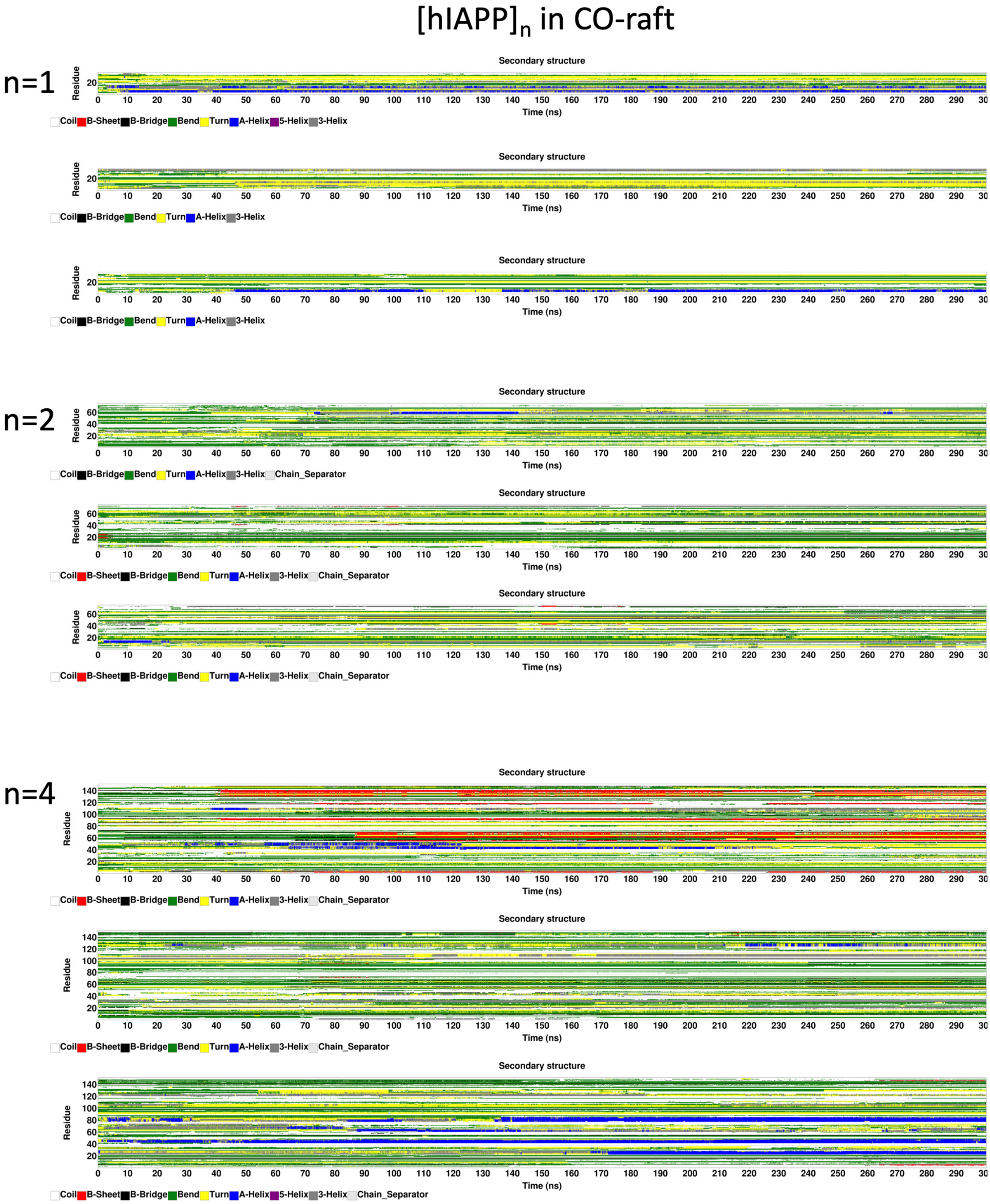

Figure SF1. Protein secondary structure of IAPP aggregates of different sizes, n = 1 (monomer), n = 2, (dimer), and n = 4 (tetramer), as a function of time (vertical axis) and simulation time (horizontal axis) in CO-raft in a DSSP plot format.

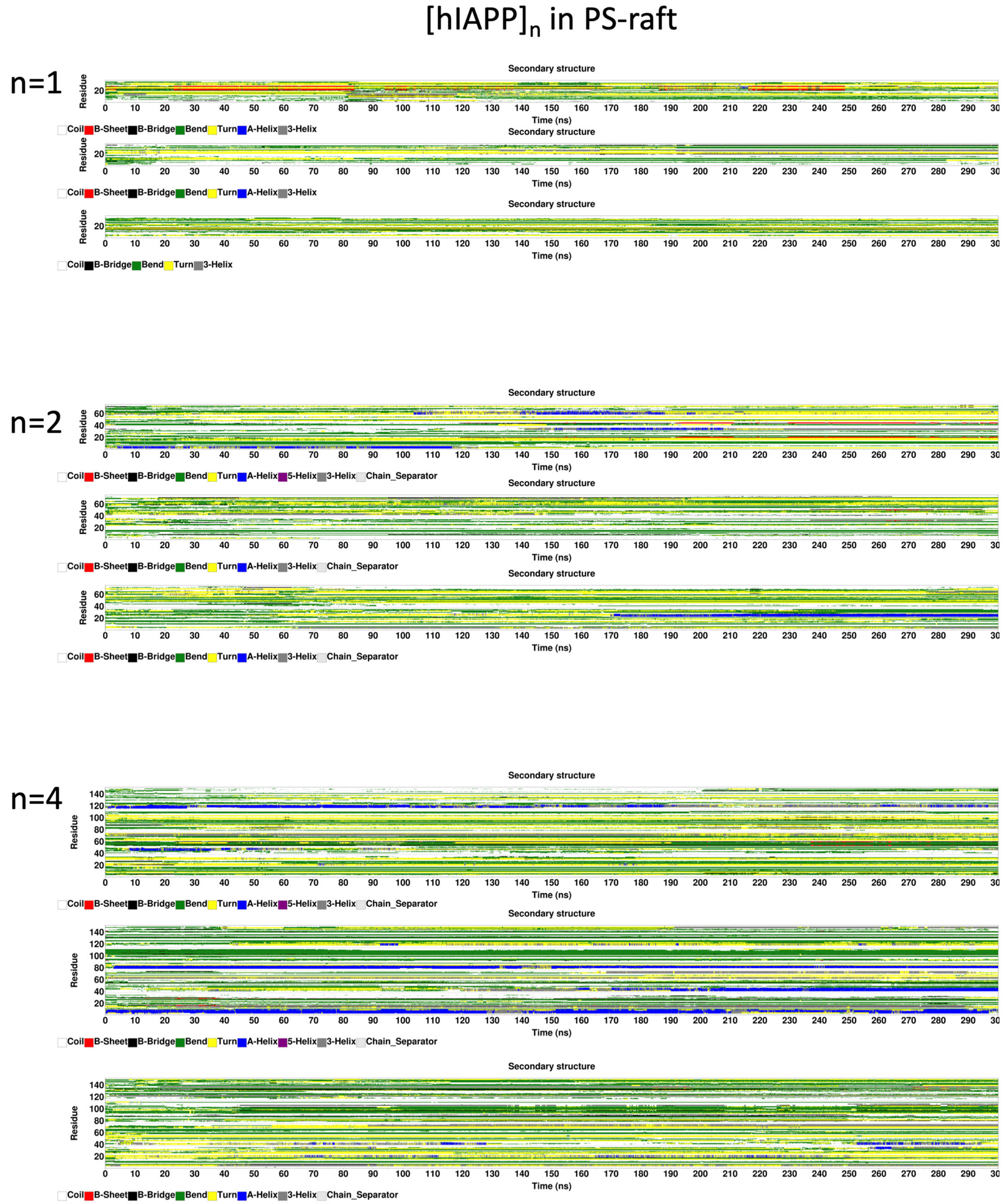

Figure SF2. Protein secondary structure of IAPP aggregates of different sizes, $n = 1$ (monomer), $n = 2$, (dimer), and $n = 4$ (tetramer), as a function of time (vertical axis) and simulation time (horizontal axis) in PS-raft in a DSSP plot format.

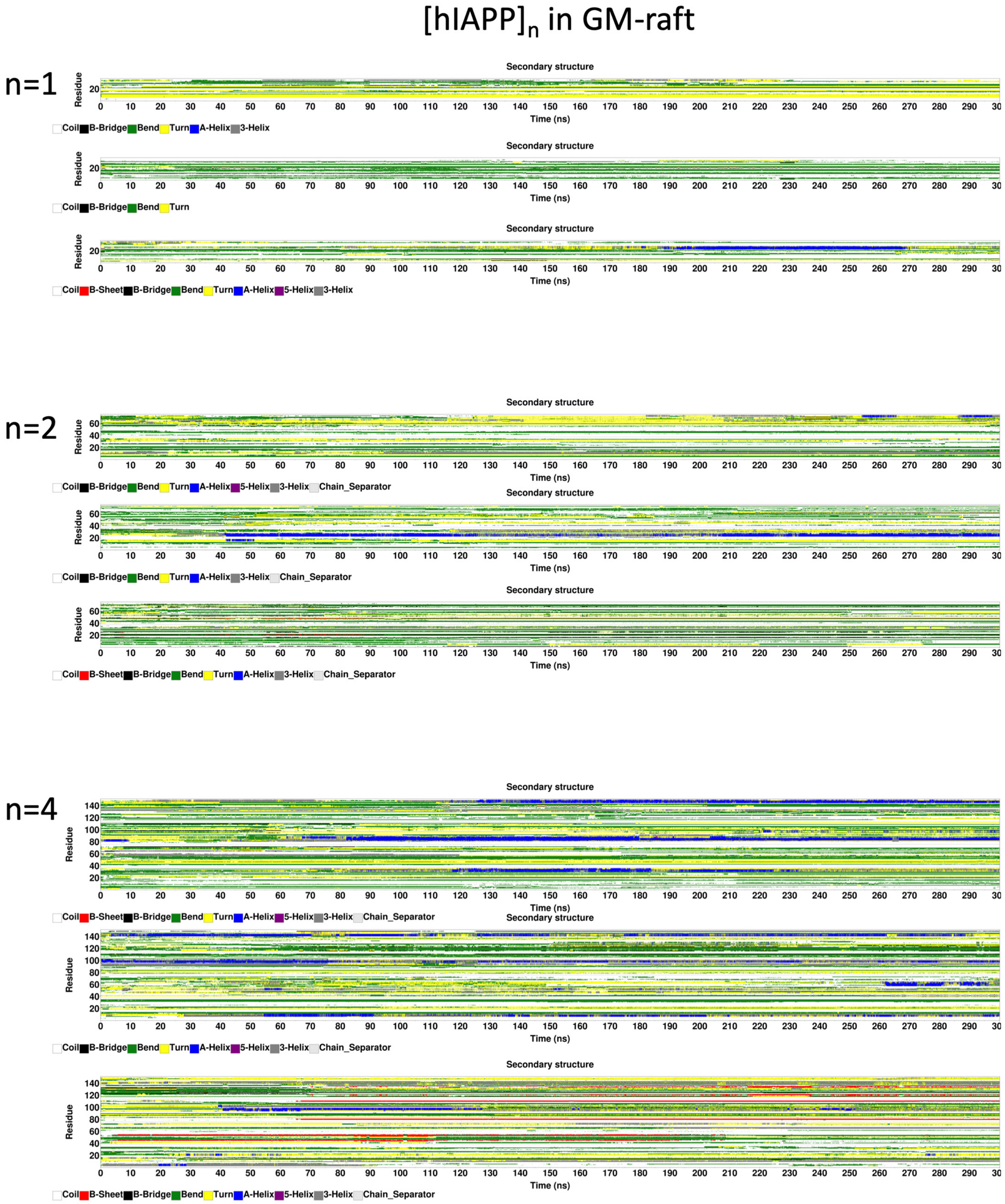

Figure SF3. Protein secondary structure of IAPP aggregates of different sizes, *n* = 1 (monomer), *n* = 2, (dimer), and *n* = 4 (tetramer), as a function of time (vertical axis) and simulation time (horizontal axis) in GM-raft in a DSSP plot format.

SI - G

Protein folding kinetics of hIAPP of hIAPP aggregates in CO-raft (Figure SG1), PS-raft (Figure SG2), and GM-raft (Figure SG3).

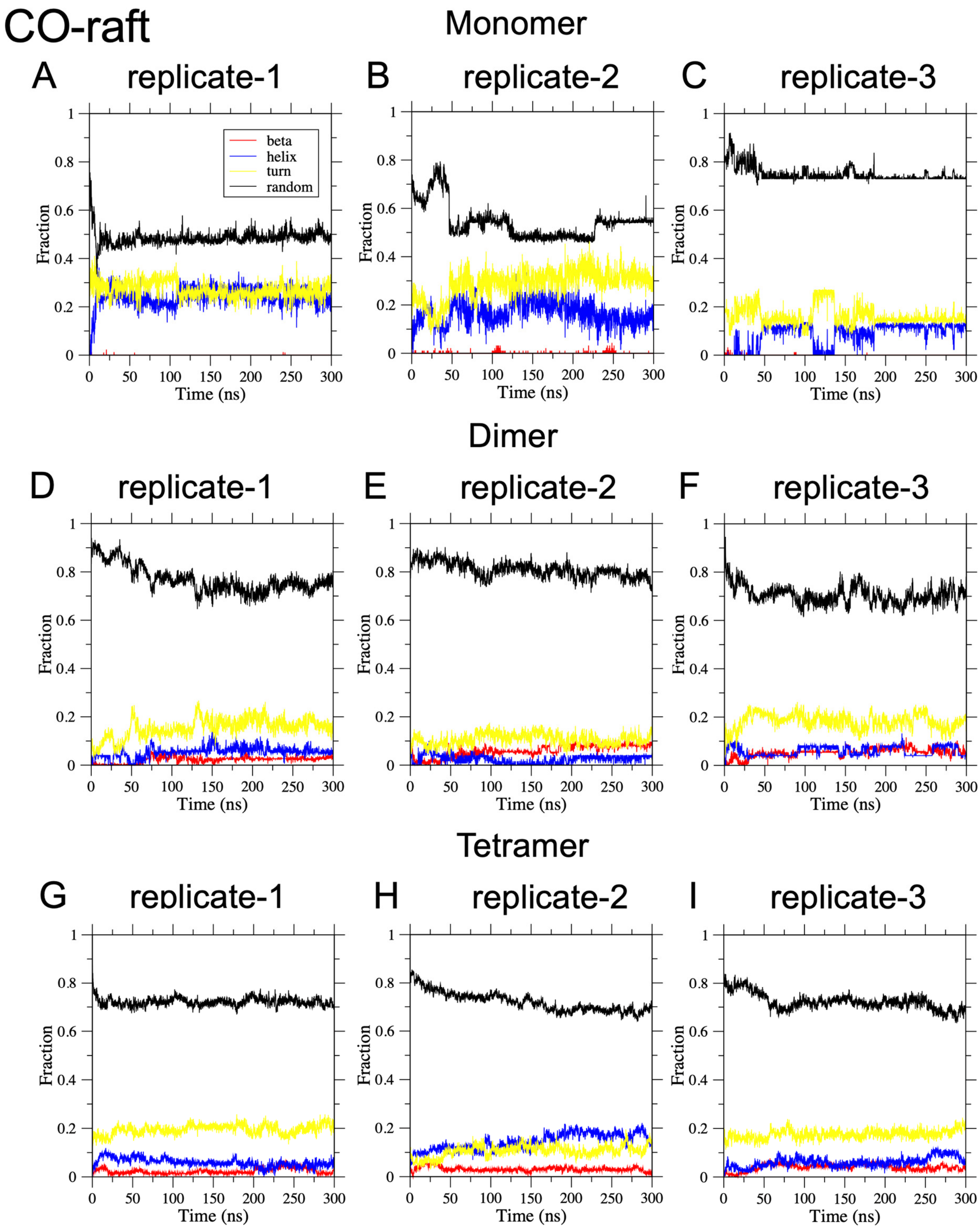

Figure SG1. The fraction of residues participated in combined secondary structures, beta (red), helix (blue), turn (yellow), and random (black), vs. time for each simulation replicates (1-3) of hIAPP aggregates of different sizes, monomer (**A-C**), dimer (**D-F**), and tetramer (**G-I**), in CO-raft.

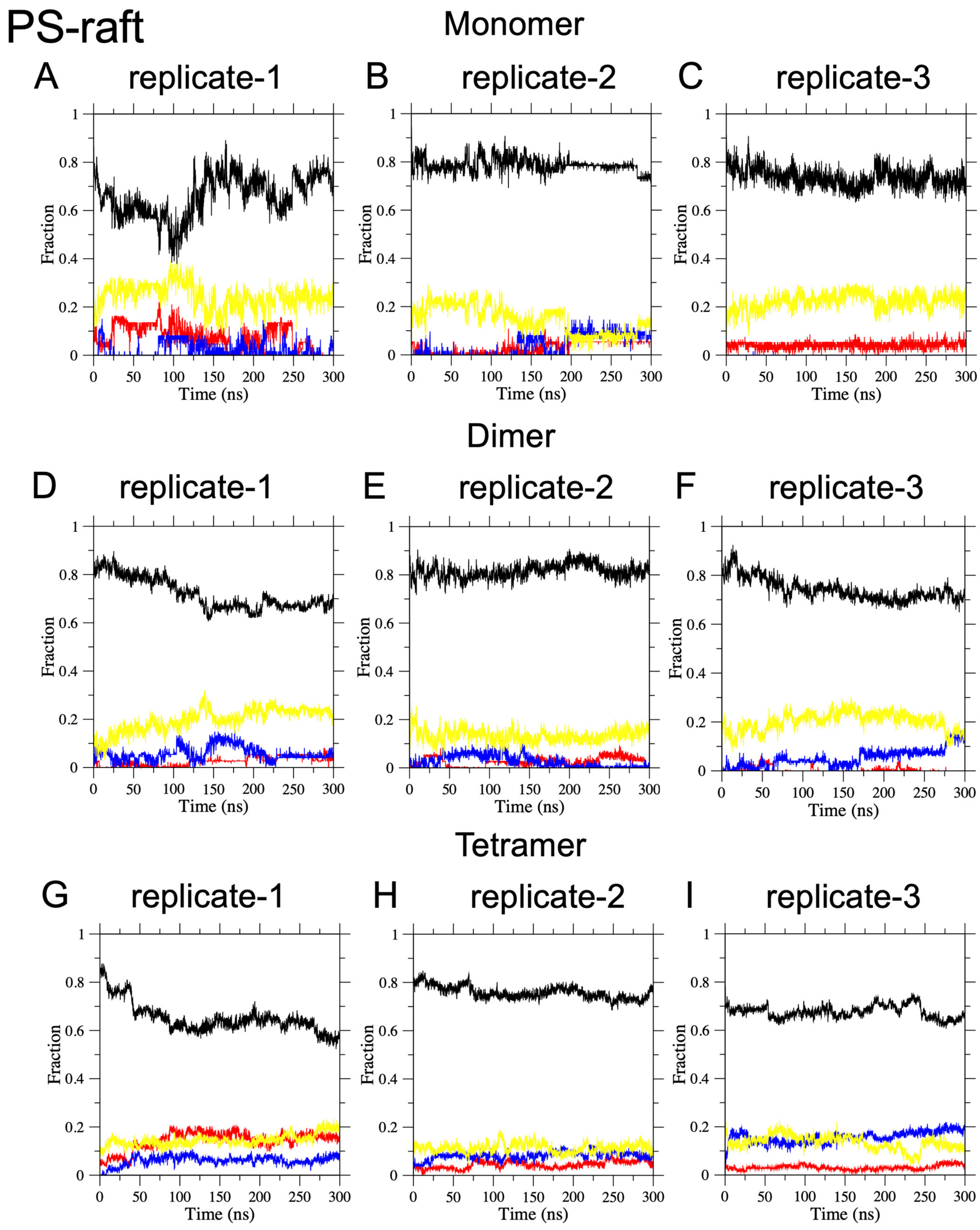

Figure SG2. The fraction of residues participated in combined secondary structures, beta (red), helix (blue), turn (yellow), and random (black), vs. time for each simulation replicates (1-3) of hIAPP aggregates of different sizes, monomer (**A-C**), dimer (**D-F**), and tetramer (**G-I**), in PS-raft.

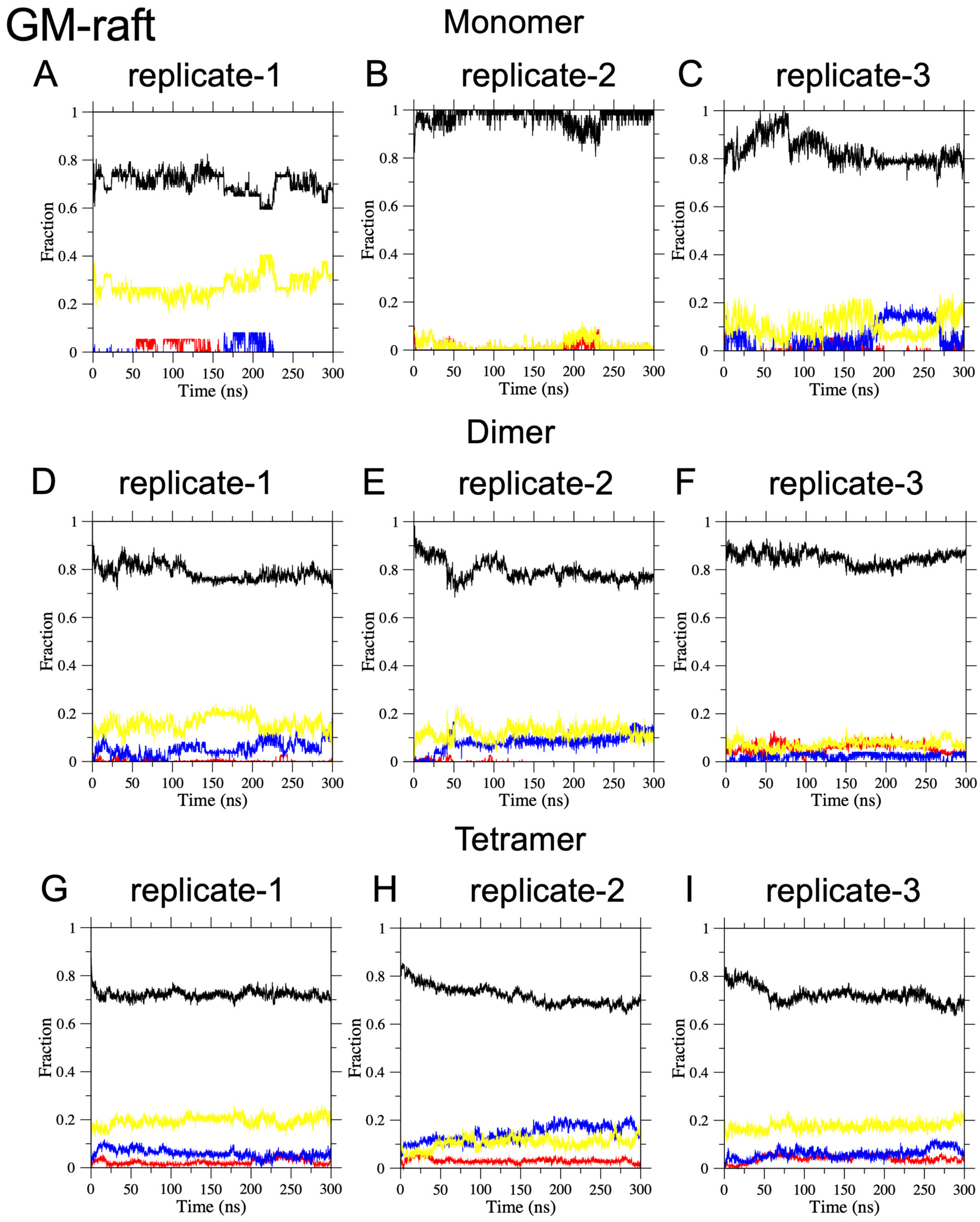

Figure SG3. The fraction of residues participated in combined secondary structures, beta (red), helix (blue), turn (yellow), and random (black), vs. time for each simulation replicates (1-3) of hIAPP aggregates of different sizes, monomer (**A-C**), dimer (**D-F**), and tetramer (**G-I**), in GM-raft.
